# Multi-locus genome-wide association mapping for spike-related traits in bread wheat (*Triticum aestivum* L.)

**DOI:** 10.1186/s12864-021-07834-5

**Published:** 2021-08-05

**Authors:** Parveen Malik, Jitendra Kumar, Shiveta Sharma, Rajiv Sharma, Shailendra Sharma

**Affiliations:** 1 Department of Genetics and Plant Breeding, ChaudharyCharan Singh University (CCSU), Meerut, 250 004 India; 2 National Agri-Food Biotechnology Institute (NABI), Sector 81(Knowledge City), SahibzadaAjit Singh Nagar, Punjab 140306 India; 3 Scotland’s Rural College (SRUC), Peter Wilson Building, West Mains Road, Edinburgh, EH9 3JG UK

**Keywords:** *Triticum aestivum* L., Molecular marker, Spike-layer uniformity, Multi-locus GWAS, Candidate genes

## Abstract

**Background:**

Bread wheat (*Triticum aestivum* L.) is one of the most important cereal food crops for the global population. Spike-layer uniformity (the consistency of the spike distribution in the vertical space)-related traits (SLURTs) are quantitative and have been shown to directly affect yield potential by modifying the plant architecture. Therefore, these parameters are important breeding targets for wheat improvement. The present study is the first genome-wide association study (GWAS) targeting SLURTs in wheat. In this study, a set of 225 diverse spring wheat accessions were used for multi-locus GWAS to evaluate SLURTs, including the number of spikes per plant (NSPP), spike length (SL), number of spikelets per spike (NSPS), grain weight per spike (GWPS), lowest tiller height (LTH), spike-layer thickness (SLT), spike-layer number (SLN) and spike-layer uniformity (SLU).

**Results:**

In total, 136 significant marker trait associations (MTAs) were identified when the analysis was both performed individually and combined for two environments. Twenty-nine MTAs were detected in environment one, 48 MTAs were discovered in environment two and 59 MTAs were detected using combined data from the two environments. Altogether, 15 significant MTAs were found for five traits in one of the two environments, and four significant MTAs were detected for the two traits, LTH and SLU, in both environments i.e. E1, E2 and also in combined data from the two environments. In total, 279 candidate genes (CGs) were identified, including Chaperone DnaJ, ABC transporter-like, AP2/ERF, SWEET sugar transporter, as well as genes that have previously been associated with wheat spike development, seed development and grain yield.

**Conclusions:**

The MTAs detected through multi-locus GWAS will be useful for improving SLURTs and thus yield in wheat production through marker-assisted and genomic selection.

**Supplementary Information:**

The online version contains supplementary material available at 10.1186/s12864-021-07834-5.

## Background

Bread wheat (*Triticum aestivum* L.) is one of the most widely grown cereal crops in the world and serves as the main energy source for approximately one-third of the global population [1–3]. Several grain yield parameters play important roles in improving wheat yield, including spike number per plant (SNPP), spike length (SL), grain weight per spike (GWPS), fertile spikelet number (FSN), spikelet number (SN), sterile spikelet number (SSN) and grain number per spike (GNPS). These parameters are important breeding targets for wheat yield improvements [4–8]. Understanding the physiological, genetic and developmental basis of spike morphology is significant not only for increasing spikelet number but also for exploiting the fruiting or grain setting productivity of spikelets [9]. Moreover, characteristics of the spike-layer uniformity (SLU) are usually determined by the variation of spike heights between inter-plants and between inter-tillers. These two indexes of the population uniformity are used not only in wheat but also in rice [10–12]. All these factors are affected by environmental factors, agronomic management and genotypes [10, 13].

The most important step for the genetic improvement of wheat is conventional wheat breeding, which is based on phenotypic selection [14]. Breeders usually increase wheat yield by modifying the spike number/hectare, grain number/ear, or 1000 grain weight [15, 16]. However, due to the high cost of generating these traits over large germplasm and the labour involved, the improvement of wheat yield using conventional breeding of spike traits is difficult. Furthermore, these traits are significantly influenced by several other factors, such as genotypic factors, environmental factors and the gene-environment interaction (G X E) [16]. Previously, three important approaches were suggested for increasing the spike size in wheat: (i) increasing spikelet number per spike [17–20]; (ii) increasing the number of florets and/or grains and grain size per spikelet [7, 21–23]; and (iii) simultaneously increasing the spikelet and floret/grain number and grain size. However, combined improvement of these traits is difficult through conventional breeding, as they are often negatively linked to each other. Marker-assisted recurrent selection (MARS) and marker-assisted backcrossing (MABC) are good options. Hence, discovering the important single nucleotide polymorphisms (SNPs)/quantitative trait loci (QTLs) associated with spike-related traits is an urgent task for wheat breeding programs.

Association mapping (AM) was combined with linkage disequilibrium (LD) to identify the function of linked markers and genes for disease-associated loci in humans [24]. Currently, it is also widely used in plants, including cereals [25, 26]. Likewise, through GWAS, marker-trait associations (MTAs) have been identified for yield and its related traits [27, 28]. However, limited efforts have been made in researching the genetics of spike-related traits for wheat. Additionally, only a few studies involving multi-locus and multi-trait GWA analyses have been conducted in wheat in general [29, 30]. There are few studies focussing on wheat yield and quality traits using multi-locus GWAS; therefore, more research is needed to overcome the limitations of routine single-locus single-trait GWAS. In recent years, several molecular mapping studies of spike-related traits have been conducted that led to the identification of QTLs in different wheat germplasms [16, 31–37], as well as multiple genes that have been identified in wheat for spike morphology traits [18–20, 22, 38, 39]. To the best of our knowledge, only a single QTL mapping study has been conducted in wheat for SLURTs involving a RIL population [12].

The present study aimed to identify MTAs involving spike-related traits using LD-based multi-locus GWA mapping using SNP markers in an association panel comprising of 225 spring wheat accessions. Some novel aspects have been included in this study and will be discussed below. First, we included the congruence of our results with historical context which reveals the genes identified for plant architecture and thus identifies the possible genes that could be targeted for yield improvements using SLURT traits. We have also highlighted the novel genes identified in this study using the multi-locus GWAS method. Additionally, we determined that these traits are not independent of the grain traits via providing the grain yield trait correlations from our unpublished data. Finally, we included genomic selection to demonstrate the moderate to high levels of efficiency in predicting SLURTs traits from the identified molecular markers, highlighting the pre-breeding relevance of the genetic markers identified. In this study, the wheat association mapping panel was tested for two years in spring-sown conditions (2017–18 and 2018–19), and phenotypic data were collected for eight different SLURTs (Table 1; Additional File 1: Fig. S1**)**. Candidate genes (CGs) underlying some of the MTAs were also identified. The MTAs identified in this study will be useful for marker-assisted selection (MAS) in the development of new desirable wheat varieties with an ideal spike-layer distribution.

**Table 1:** List of eight studied traits and their abbreviations

Trait (unit)	Abbreviation	Description
Number of Spikes Per Plant (number)	NSPP	Total numbers of normal spikes were counted from five plants of each plot.
Spike Length (cm)	SL	It measured from the base of the spike to the tip, excluding awns (only main tillers were used and five values per plots averaged to obtain the SL values).
Number of Spikelets Per Spike (number)	NSPS	A total of five random spikes from plants selected for NSPP were used for this measurement, spikelet number was counted from the basal sterile spikelet to the top fertile spikelet.
Grain Weight Per Spike (gm)	GWPS	After thrashing and cleaning of five spikes, the grain weight was calculated which was based on the mean of five spike per plot.
Lowest Tiller Height (cm)	LTH	Measured from the ground level to the tip of the spike and excluding awns.
Spike Layer Thickness (cm)	SLT	It measured how the spikes are distributed in a plant and measured the dispersion of the inter-tiller height. Measured as PH-LTH + SL.
Spike Layer Number (number)	SLN	Ratio of SLT and SL (SLT/SL).
Spike Layer Uniformity (ratio)	SLU	It measures the uniformity of the spikes of the inter-tiller of a genotype or the consistency of the spike distribution in the vertical space and calculated as SLU=SL/SLT. A value of 1 indicates that all the inter-tiller spike heights are identical. However, lower values indicate the uneven heights of the measured spikes of the genotype.

Note: Plant height (PH) was measured (in cm) from the ground level to the tip of the spike excluding awns

## Results

### Phenotypic variation and statistical analysis

The ANOVA test revealed significant differences in the measured traits with the following sources of variation: genotypes (G), environments (E), G X E interaction and genotype within replications. Except for LTH, all traits displayed significant variation between the factors, indicating environment-specific effects on measuring these traits, the F-values of each trait are given in Table 2. Thus, to capture the effects of each environment, GWAS analysis was performed on each environment individually along with combined analysis. Estimates of broad-sense heritability (*H*^2^) of these traits were generally moderate to high and ranged from 39% (SLN) to 91% (GWPS), indicating the robustness of the measured traits (Table 2).

**Table 2:** Analysis of variance (ANOVA) of eight spike related traits tested for two environments

Source of variation	DF	F value							
		NSPP	SL	NSPS	GWPS	LTH	SLT	SLN	SLU
Environment	1	50.42***	67.24***	16.89***	1.29	1.29	89.49***	60.84***	91.95***
Genotype	224	3.49***	11.85***	9.03***	10.76***	10.76***	1.98***	1.88***	3.17***
Rep with in ENV	1	1.61	4.60**	0.01	4.78*	4.78*	36.18***	39.02***	8.24***
Genotype X ENV	224	8.70***	6.08***	5.81***	5.62***	1.18	1.30*	1.23*	2.05***
Heritability		69%	76%	89%	91%	84%	40%	39%	53%

*DF*: Degree of freedom, *ENV*: Environment, Rep: Replication, *NSPP*: Number of spike per plant, *SL*: Spike length, *NSPS*: Number of spikelets per spike, *GWPS*: Grain weight per spike, *LTH*: Lowest tiller height, *SLT*: Spike-layer thickness, *SLN*: Spike-layer number, *SLU*: Spike-layer uniformity, ***; 0.001, **; 0.01, *; 0.05 level of significance

The pairwise correlation coefficient (r) analysis of the eight spike-related traits in individual environments, as well as the combined data from both environments (E1 and E2) revealed strong correlations among several measured traits (Additional file 1 Fig. S2, S3, S4). Correlation analysis was also performed for spike-related traits and grain yield (GYPP) from data made available from our unpublished study. Fifty-one significant correlation combinations were found between different traits including grain yield in E1, E2 and the combined data, including 19 in E1 (11 positive and 8 negative) and 15 in E2 (8 positive and 7 negative) and 17 in the combined data (10 positive and 7 negatives). The SLU was negatively correlated with SLN and SLT in E1, E2 and the combined data. SLU was also negatively correlated with grain yield. The correlation between the SLN and SLT was positive in E1, E2 and the combined data. Positive correlations (*P* < 0.05) were observed between SLN and GYPP for E1 (Additional file 1; Fig. S2) and between SLT and GYPP for E1 and the combined data (Additional file 1; Fig. S2, S4).

### Population structure and linkage disequilibrium (LD) analysis

**Fig. 1:**
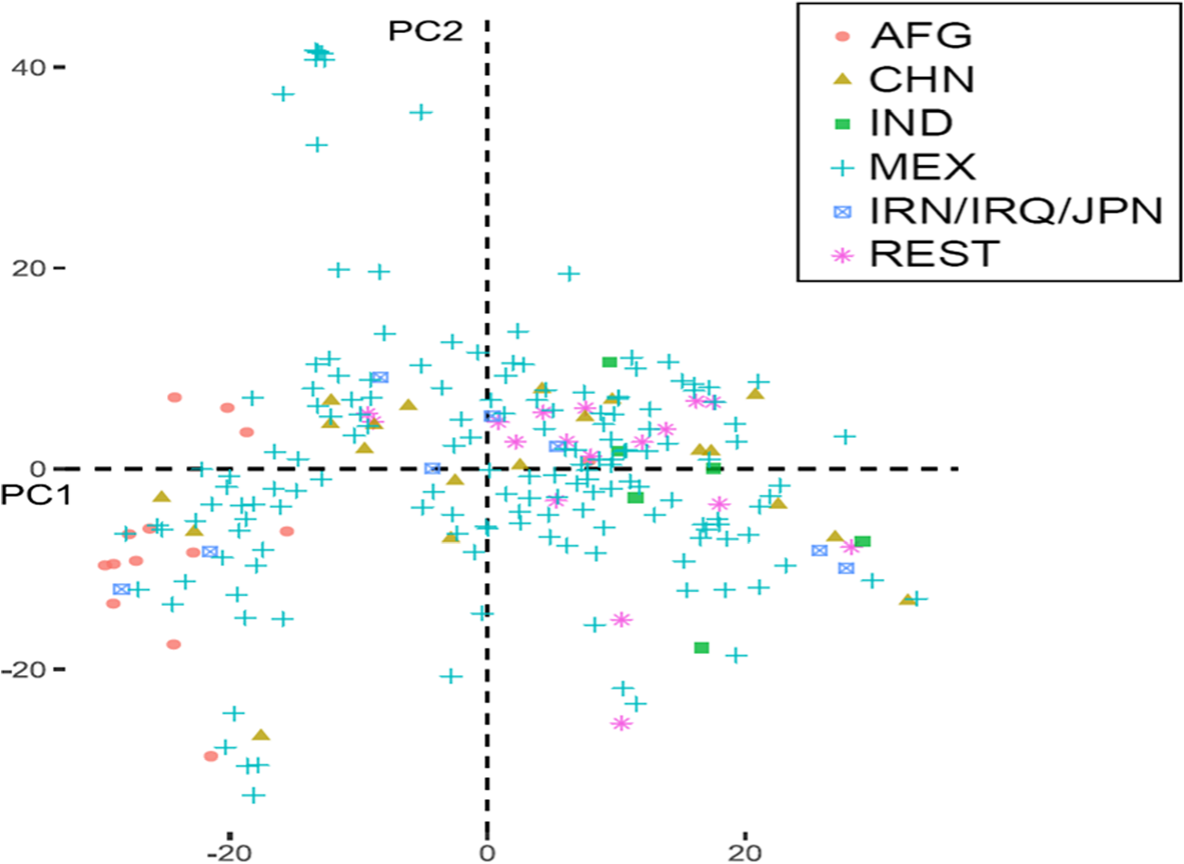
Principal component analysis (PCA) obtained using polymorphic SNP markers showing the distribution of 225 SWRS wheat genotypes along with the two components. PC1 explained 7% while PC2 explained 4% variance

The PCA results illustrate the population structure of the studied population (Fig. 1). Accessions originating from Afghanistan clustered together and formed clusters along the PC1 (towards the right). Accessions from Mexico were distributed almost everywhere. Furthermore, accessions from India and China were also grouped on the PC1 axis. LD decay distance ranged from 2 cM to 20 cM in different genomic regions (30).

### Marker–trait associations (MTAs)

A total of 136 significant MTAs were detected for the eight traits in the individual environments and the combined data with (−log10 [*p*-value] ≥ 3). In environment one (E1), 29 significant MTAs were detected for eight traits, with five for GWPS, seven for LTH, four for NSPP, two for each NSPS & SL, one for each SLN & SLT and seven for SLU. The 29 MTAs were distributed on 11 different chromosomes. In environment two (E2), 48 significant MTAs were found for the eight traits, with three for GWPS, five for LTH, six for NSPP, seven for NSPS, two for SL, seven for SLN, eight for SLT and 10 for SLU. These 48 MTAs were distributed on 14 different chromosomes. In the combined data analysis, 59 significant MTAs were identified for the eight traits, with five each for GWPS & LTH, seven each for NSPP & NSPS, five for SL, 13 for SLN, three for SLT and 14 for SLU. These MTAs were mapped on 18 different chromosomes (Table 3). Altogether, 15 common significant MTAs were found for five traits in either of the two environments. Four MTAs (3 associated with LTH and 1 associated with SLU) were detected in both environments i.e. E1, E2 and also in combined data from the two environments. Fourteen MTAs were common between E2 and the combined data (E1 and E2). A single MTA was common between E1 and the combined data (E1 and E2). Details of all these MTAs are presented in Table 4.

**Table 3:** List of significant SNP markers (−log10 [p-value] ≥ 3) associated with different spike related traits using the individual environment and combined data

Environment 1 (E1; 2017–18)						Environment 2 (E2; 2018–19)						Combined data (E1; 2017–18 and E2; 2018–19)					
SNP	Id	Chr.	Pos.	P.value	Effect	SNP	Id	Chr.	Pos.	P.value	Effect	SNP	Id	Chr.	Pos.	P.value	Effect
**GWPS**						**GWPS**						**GWPS**					
SNP_10127	1,094,714	5B	102	3.24E-04	0.84	SNP_109	980,521	5A	169	2.29E-04	0.82	SNP_5177	1,089,342	2D	25	4.09E-04	1.11
SNP_493	3,025,448	2B	156	5.57E-04	1.09	SNP_1671	1,011,353	6B	74	3.52E-04	0.83	SNP_493	3,025,448	2B	156	4.46E-04	1.11
SNP_5177	1,089,342	2D	25	5.97E-04	1.07	SNP_8417	1,076,441	2A	222	9.50E-04	1.33	SNP_109	980,521	5A	169	6.93E-04	0.68
SNP_8783	1,023,982	3A	18	7.99E-04	0.78	**LTH**						SNP_7209	1,006,397	4A	177	7.13E-04	0.76
SNP_5621	1,241,685	2B	155	9.91E-04	0.73	SNP_2448	1,059,739	1A	127	5.72E-05	−7.77	SNP_13995	1,228,280	1A	76	8.15E-04	0.92
**LTH**						SNP_2970	1,009,860	1A	175	4.29E-04	−4.29	**LTH**					
SNP_1852	982,036	6A	175	9.09E-05	−3.24	SNP_12067	1,147,118	2A	132	8.93E-04	−5.51	SNP_2448	1,059,739	1A	127	8.05E-05	−6.15
SNP_8107	1,091,955	6A	173	1.88E-04	−3.35	SNP_1852	982,036	6A	175	9.07E-04	− 3.57	SNP_1800	3,025,720	6A	159	1.21E-04	−3.76
SNP_1869	1,093,162	6A	175	1.88E-04	−3.42	SNP_1800	3,025,720	6A	159	9.36E-04	3.91	SNP_1852	982,036	6A	175	4.73E-04	−3.03
SNP_2448	1,059,739	1A	127	6.60E-04	−4.53	**NSPP**						SNP_2933	1,091,284	6A	175	7.03E-04	−3.42
SNP_2933	1,091,284	6A	175	6.90E-04	−2.9	SNP_11101	1,011,157	3B	104	1.26E-04	−0.67	SNP_2970	1,009,860	1A	175	7.84E-04	−4.5
SNP_1800	3,025,720	6A	159	7.01E-04	−2.49	SNP_454	1,001,462	4B	64	2.09E-04	0.56	**NSPP**					
SNP_5334	982,125	7B	184	7.34E-04	−2.71	SNP_10510	1,079,112	7A	304	2.18E-04	− 0.68	SNP_454	1,001,462	4B	64	1.04E-04	− 0.41
**NSPP**						SNP_4595	1,090,297	7B	70	5.69E-04	−0.63	SNP_4595	1,090,297	7B	70	2.91E-04	−0.34
SNP_6294	1,006,701	6A	33	6.69E-06	0.41	SNP_5062	3,064,849	7B	40	6.51E-04	0.38	SNP_11101	1,011,157	3B	104	3.63E-04	−0.38
SNP_2495	1,066,071	3B	298	5.34E-04	0.26	SNP_41	1,010,420	7B	134	9.18E-04	−0.39	SNP_11135	1,129,086	5B	106	4.14E-04	−0.36
SNP_13287	1,090,849	3B	156	7.46E-04	−0.21	**NSPS**						SNP_13287	1,090,849	3B	156	6.23E-04	0.37
SNP_598	1,106,300	2A	160	8.30E-04	0.28	SNP_4596	1,090,518	6A	183	4.78E-05	1.17	SNP_10864	1,262,277	4A	227	7.08E-04	0.3
**NSPS**						SNP_4839	1,077,393	2B	151	1.21E-04	1.09	SNP_10510	1,079,112	7A	304	7.54E-04	−0.3
SNP_4528	3,024,470	2B	164	3.03E-04	−0.49	SNP_13896	1,167,029	2B	164	2.19E-04	1.12	**NSPS**					
SNP_1779	991,804	2A	89	4.31E-04	−0.48	SNP_813	1,087,368	1A	127	3.45E-04	1.23	SNP_813	1,087,368	1A	127	5.24E-05	0.89
**SL**						SNP_1766	1,077,298	6A	193	4.14E-04	0.74	SNP_1779	991,804	2A	89	3.19E-04	0.94
SNP_10587	992,268	3A	118	3.63E-04	−0.36	SNP_10039	1,058,522	5A	71	7.82E-04	0.74	SNP_13896	1,167,029	2B	164	3.26E-04	0.83
SNP_12221	1,002,874	6B	74	9.90E-04	−0.31	SNP_12996	1,067,398	2B	55	9.23E-04	−0.87	SNP_12996	1,067,398	2B	55	3.50E-04	−0.71
**SLN**			0			**SL**						SNP_4596	1,090,518	6A	183	4.00E-04	0.56
SNP_241	985,588	5B	115	9.15E-04	0.29	SNP_12504	1,091,267	4A	107	5.10E-05	0.49	SNP_1766	1,077,298	6A	193	4.42E-04	−0.53
**SLT**						SNP_9403	3,027,359	7A	112	1.40E-04	0.41	SNP_5026	1,130,302	6A	71	6.36E-04	0.56
SNP_1870	981,078	5A	27	7.06E-04	2.21	**SLN**						**SL**					
**SLU**						SNP_11184	1,120,035	1B	277	3.32E-05	−0.53	SNP_3859	1,192,931	7B	135	3.17E-04	0.27
SNP_5177	1,089,342	2D	25	1.64E-04	0.06	SNP_13306	1,128,418	6B	51	8.08E-05	−0.4	SNP_5909	1,040,130	6D	185	3.95E-04	−0.26
SNP_1870	981,078	5A	27	2.29E-04	−0.05	SNP_13877	1,206,584	6B	65	8.88E-05	−0.42	SNP_317	985,312	4B	58	5.37E-04	0.31
SNP_11011	3,064,643	5A	21	3.03E-04	−0.04	SNP_9637	1,227,916	3B	227	1.07E-04	−0.55	SNP_12504	1,091,267	4A	107	5.78E-04	0.31
SNP_10789	1,229,353	5A	29	5.46E-04	0.04	SNP_2980	1,118,663	2B	149	4.15E-04	−0.45	SNP_7958	1,211,711	6B	60	7.71E-04	0.46
SNP_10924	3,064,834	5B	102	5.83E-04	0.04	SNP_4799	3,064,625	5B	106	5.39E-04	0.25	**SLN**					
SNP_14151	1,090,816	6A	1	6.88E-04	−0.03	SNP_3408	3,064,625	5B	106	6.90E-04	0.25	SNP_10880	1,210,090	1A	445	3.44E-08	0.21
SNP_3494	1,218,597	6A	167	9.25E-04	0.04	**SLT**						SNP_11184	1,120,035	1B	277	4.73E-08	−0.38
–	–	–	–	–	–	SNP_491	1,111,091	1B	451	3.50E-05	−3.68	SNP_13296	1,129,001	1D	231	1.01E-04	0.23
–	–	–	–	–	–	SNP_1105	1,093,432	1B	187	3.78E-05	−3.27	SNP_5061	1,126,012	2B	106	1.10E-04	0.15
–	–	–	–	–	–	SNP_11184	1,120,035	1B	277	4.08E-05	−3.44	SNP_1140	987,653	3B	117	1.14E-04	−0.16
–	–	–	–	–	–	SNP_1784	1,032,134	1B	262	6.88E-05	−2.63	SNP_8087	1,149,694	3D	292	1.46E-04	−0.15
–	–	–	–	–	–	SNP_2980	1,118,663	2B	149	2.24E-04	−3.64	SNP_13268	1,003,566	4A	242	1.89E-04	0.14
–	–	–	–	–	–	SNP_11490	1,094,450	3B	24	2.69E-04	3.07	SNP_1208	987,611	5A	161	2.15E-04	−0.15
–	–	–	–	–	–	SNP_6814	1,000,905	3B	34	8.27E-04	−2.81	SNP_4799	3,064,625	5B	106	2.53E-04	0.13
–	–	–	–	–	–	SNP_13306	1,128,418	6B	51	8.62E-04	−1.75	SNP_3886	3,064,747	5B	102	3.93E-04	0.1
–	–	–	–	–	–	**SLU**						SNP_1091	1,206,846	6A	103	4.07E-04	−0.24
–	–	–	–	–	–	SNP_11184	1,120,035	1B	277	7.32E-05	0.06	SNP_10537	1,127,855	6B	153	4.20E-04	−0.15
–	–	–	–	–	–	SNP_5004	1,096,126	3B	161	1.41E-04	0.08	SNP_5909	1,040,130	6D	185	4.22E-04	−0.12
–	–	–	–	–	–	SNP_10924	3,064,834	5B	102	1.47E-04	0.1	**SLT**					
–	–	–	–	–	–	SNP_4799	3,064,625	5B	106	1.89E-04	0.06	SNP_11184	1,120,035	1B	277	7.18E-05	−2.81
–	–	–	–	–	–	SNP_3408	3,064,625	5B	106	2.43E-04	−0.05	SNP_4799	3,064,625	5B	106	2.22E-04	1.48
–	–	–	–	–	–	SNP_13306	1,128,418	6B	51	4.17E-04	0.07	SNP_3408	3,064,625	5B	106	3.13E-04	1.44
–	–	–	–	–	–	SNP_5327	998,729	6D	186	4.73E-04	−0.05	**SLU**					
–	–	–	–	–	–	SNP_10576	1,130,017	6D	187	8.58E-04	0.06	SNP_1792	1,209,199	1A	280	3.05E-08	0.04
–	–	–	–	–	–	SNP_8090	1,130,017	6D	187	8.59E-04	−0.05	SNP_11184	1,120,035	1B	277	9.56E-07	0.05
–	–	–	–	–	–	SNP_5909	1,040,130	6D	185	9.61E-04	0.05	SNP_8940	1,207,869	3A	201	2.01E-05	−0.03
–	–	–	–	–	–	–	–	–	–	–	–	SNP_2821	1,321,784	3B	162	5.84E-05	0.02
–	–	–	–	–	–	–	–	–	–	–	–	SNP_3884	1,002,975	3B	254	1.04E-04	−0.02
–	–	–	–	–	–	–	–	–	–	–	–	SNP_9866	1,256,184	3B	133	1.06E-04	0.02
–	–	–	–	–	–	–	–	–	–	–	–	SNP_2796	1,083,825	4A	221	1.55E-04	−0.02
–	–	–	–	–	–	–	–	–	–	–	–	SNP_6196	1,127,488	4A	39	1.61E-04	−0.02
–	–	–	–	–	–	–	–	–	–	–	–	SNP_444	992,456	4A	129	1.89E-04	0.03
–	–	–	–	–	–	–	–	–	–	–	–	SNP_218	1,042,220	4B	90	4.63E-04	−0.03
–	–	–	–	–	–	–	–	–	–	–	–	SNP_652	990,542	5A	95	5.88E-04	−0.02
–	–	–	–	–	–	–	–	–	–	–	–	SNP_10924	3,064,834	5B	102	6.04E-04	0.02
–	–	–	–	–	–	–	–	–	–	–	–	SNP_13218	1,209,264	6A	191	9.11E-04	−0.03
–	–	–	–	–	–	–	–	–	–	–	–	SNP_7430	1,127,751	7A	173	9.46E-04	0.02

*Chr*: Chromosome, *Pos*: Position (cM), *GWPS*: Grain weight per spike, *LTH*: Lowest tiller height, *NSPP*: Number of spike per plant, *SL*: Spike length, *NSPS*: Number of spikelets per spike, *SLN*: Spike-layer number, *SLT*: Spike-layer thickness, *SLU*: Spike-layer uniformity

**Table 4:** List of common significant MTAs for a particular trait in Environment 1 (E1), Environment (E2), and combined environments (E1 and E2) at *P*-value ≤0.0001

Trait	SNP	Id	Allele	Chr.	Pos.	E1 (2017–18)		E2 (2018–19)		Combined (E1 & E2)	
						P-value	Effect	P-value	Effect	P-value	Effect
GWPS	SNP_109*	980,521	C/T	5A	169	–	–	2.29E-04	0.82	6.93E-04	0.68
	SNP_493#	3,025,448	G/A	2B	156	–	–	5.57E-04	1.09	4.46E-04	1.11
LTH	SNP_1800	3,025,720	C/A	6A	159	7.01E-04	−2.49	9.07E-04	−3.57	4.73E-04	−3.03
	SNP_1852	982,036	A/C	6A	175	9.09E-05	−3.24	4.29E-04	−4.29	1.21E-04	−3.76
	SNP_2448*	1,059,739	G/A	1A	127	6.60E-04	−4.53	5.72E-05	−7.77	8.05E-05	−6.15
	SNP_2933	1,091,284	G/T	6A	175	6.90E-04	−2.90	–	–	7.03E-04	−3.42
	SNP_2970	1,009,860	C/T	1A	175	–	–	8.93E-04	−5.51	7.84E-04	−4.50
NSPP	SNP_11101	1,011,157	G/A	3B	104	–	–	1.26E-04	−0.67	4.14E-04	−0.36
	SNP_454*	1,001,462	A/G	4B	064	–	–	2.09E-04	0.56	7.08E-04	0.30
	SNP_4595	1,090,297	G/T	7B	070	–	–	5.69E-04	−0.63	3.63E-04	−0.38
NSPS	SNP_10510	1,079,112	C/T	7A	304	–	–	2.18E-04	−0.68	1.04E-04	−0.41
	SNP_12996	1,067,398	C/A	2B	055	–	–	9.23E-04	−0.87	3.50E-04	−0.71
	SNP_13896#	1,167,029	C/T	2B	164	–	–	2.19E-04	1.12	3.26E-04	0.83
	SNP_1766	1,077,298	G/A	6A	193	–	–	4.14E-04	0.74	4.00E-04	0.56
	SNP_1779#$	991,804	C/G	2A	089	–	–	4.31E-04	−0.48	4.42E-04	−0.53
	SNP_4596*$	1,090,518	T/A	6A	183	–	–	4.78E-05	1.17	5.24E-05	0.89
	SNP_813#$	1,087,368	A/G	1A	127	–	–	3.45E-04	1.23	3.19E-04	0.94
SL	SNP_12504	1,091,267	A/G	4A	107	–	–	5.10E-05	0.49	5.78E-04	0.31
SLU	SNP_10924^@^	3,064,834	A/G	5B	102	9.25E-04	0.04	1.89E-04	0.06	3.05E-08	0.04

Chr: Chromosome, Pos: Position (cM), *: MTA detected in a reported QTL interval for same traits, #: MTA detected in a reported QTL interval for grain yield, *$: MTA detected near to (< 50 Mb) reported QTL interval for same trait and #$: MTA detected near to (< 50 Mb) reported QTL interval for grain yield trait. @; MTA that qualified FDR

**Fig. 2:**
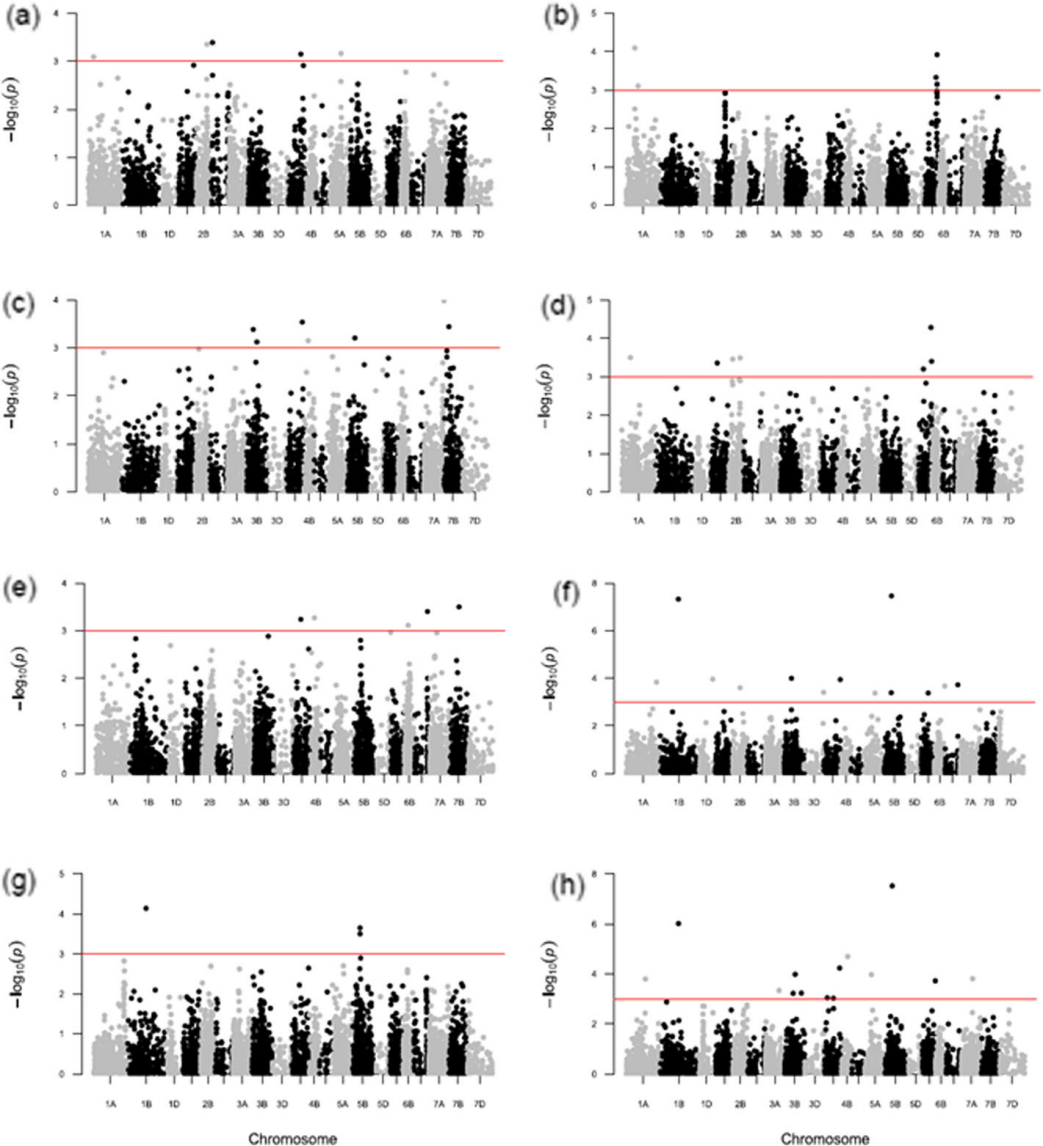
Manhattan plots obtained using FarmCPU for eight SLURTs (**a**) GWPS, (**b**) LTH, (**c**) NSPP, (**d**) NSPS, (**e**) SL, (**f**) SLN, (**g**) SLT (**h**) SLU under combined E1 and E2 conditions

**Fig. 3:**
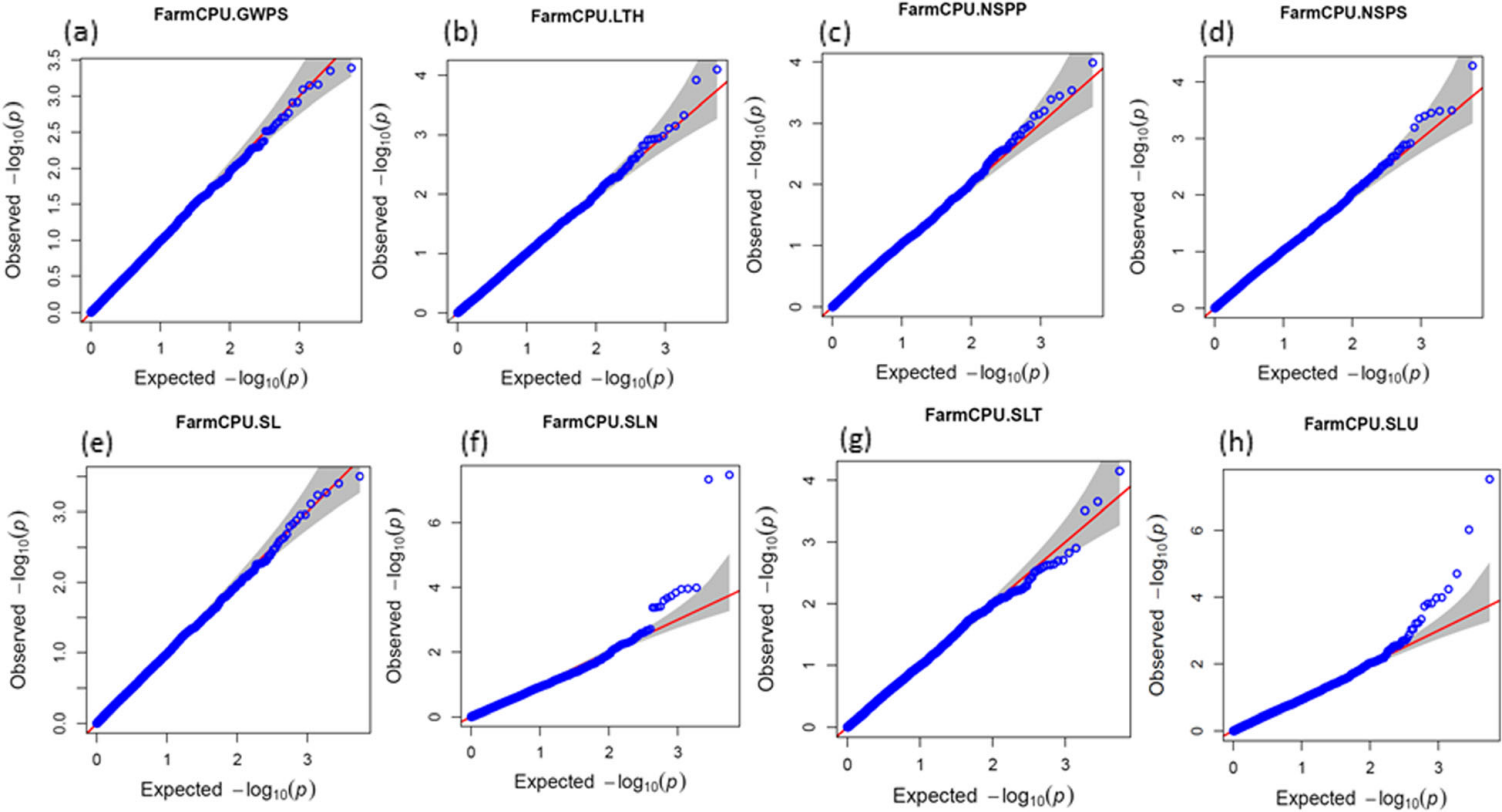
QQ-plots to visualize the deviation of observed *p* values from expected *p* values (based on null hypothesis) for eight SLURTs (**a**) GWPS, (**b**) LTH, (**c**) NSPP, (**d**) NSPS, (**e**) SL, (**f**) SLN, (**g**) SLT, (**h**) SLU under combined E1 and E2 conditions

Furthermore, 6 multi-trait MTAs were found to be associated with traits such as SLN, SLT, SLU and GWPS. The details of these MTAs are presented in Table 5. After the false discovery rate (FDR) multiple corrections, only 5 MTAs were identified. These MTAs were detected in E1, combined data and were associated with three traits only. Details of these MTAs are presented in Table 6. Manhattan plots were used to display the SNPs associated with traits. The solid horizontal line indicates the cut-off *p*-value of significant SNPs with the trait (Fig. 2 and Additional file 1; Fig. S5). Similarly, the quantile-quantile (Q-Q) plots of the observed and expected distribution of *p*-values found nearly linear trends, showing appropriate model fitting for the GWAS test, as shown in Fig. 3 and Additional file 1 and Fig. S6.

**Table 5:** Details of common significant MTAs associated with more than one trait

Traits	SNP	Id	Allele	Chr.	Pos.
SLN; E2 & C, SLT; E2 & C, SLU; E2 & C	SNP_11184	1,120,035	T/C	1B	277
SLN; E2, SLT; E2, SLU; C	SNP_13306	1,128,418	C/T	6B	51
SLT and SLU; E1	SNP_1870	981,078	T/C	5A	27
SLN; E2, SLU; E2, SLT; C	SNP_3408	3,064,625	A/G	5B	106
SLN; E2 & C, SLU; E2, SLT; C	SNP_4799	3,064,625	C/G	5B	106
GWPS; E1 & C, SLU; E1 & E2, SL; C, SLN; C	SNP_5177	1,089,342	G/A	2D	25

E1: Environment 1, E2: Environment 2, C: Combined data (E1 and E2), Chr: Chromosome, Pos: Position (cM)

**Table 6:** List of FDR qualified MTAs for three traits in environment 1 and combined data

Trait	SNP	Id	Chr.	Pos.	P-value	Corrected P-value	Effect
Environment 1 (E1; 2017–18)							
NSPP	SNP_6294	1,006,701	6A	0.33	0.00000669	0.05	0.41
Combined data (E1 and E2)							
SLN	SNP_4799	3,064,625	5B	1.06	3.44E-08	0.000332	0.21
	SNP_11184	1,120,035	1B	2.77	4.73E-08	0.000228	−0.38
SLU	SNP_10924	3,064,834	5B	1.02	3.05E-08	0.000294	0.04
	SNP_11184	1,120,035	1B	2.77	0.000000956	0.00461	0.05

Chr: chromosome, Pos: position in cM

### Comparison of MTA or MTA groups (QTLs) detected in the current study to historical QTL regions

In comparison with previously known QTL intervals, MTAs or MTA groups (QTLs) detected in the current study were detected either within or less than 50 MB of previously known QTL intervals. MTA was considered novel if it was detected at a different or faraway (> 50 MB) position. Six MTAs were found within the interval of reported QTLs for the same traits (Additional File 1: Fig. S7). These MTAs were associated with three traits: GWPS [SNP_7209; 40, and SNP_109; 41], LTH [SNP_2448; 42 and SNP_12067; 43], and NSPP [SNP_598 and SNP_454; 44]. Six MTAs were also detected in the interval of QTLs reported previously for grain yield. These MTAs were associated with three traits: GWPS (SNP_5621 and SNP_493; 43], NSPS [SNP_4528 and SNP_13896; 43] and SLN [SNP_2980 and SNP_1140; 43]. Twenty-seven MTAs were also found near the reported QTL intervals for the same traits or grain yield. Fifty three MTAs were novel.

### Genomic prediction

**Fig. 4:**
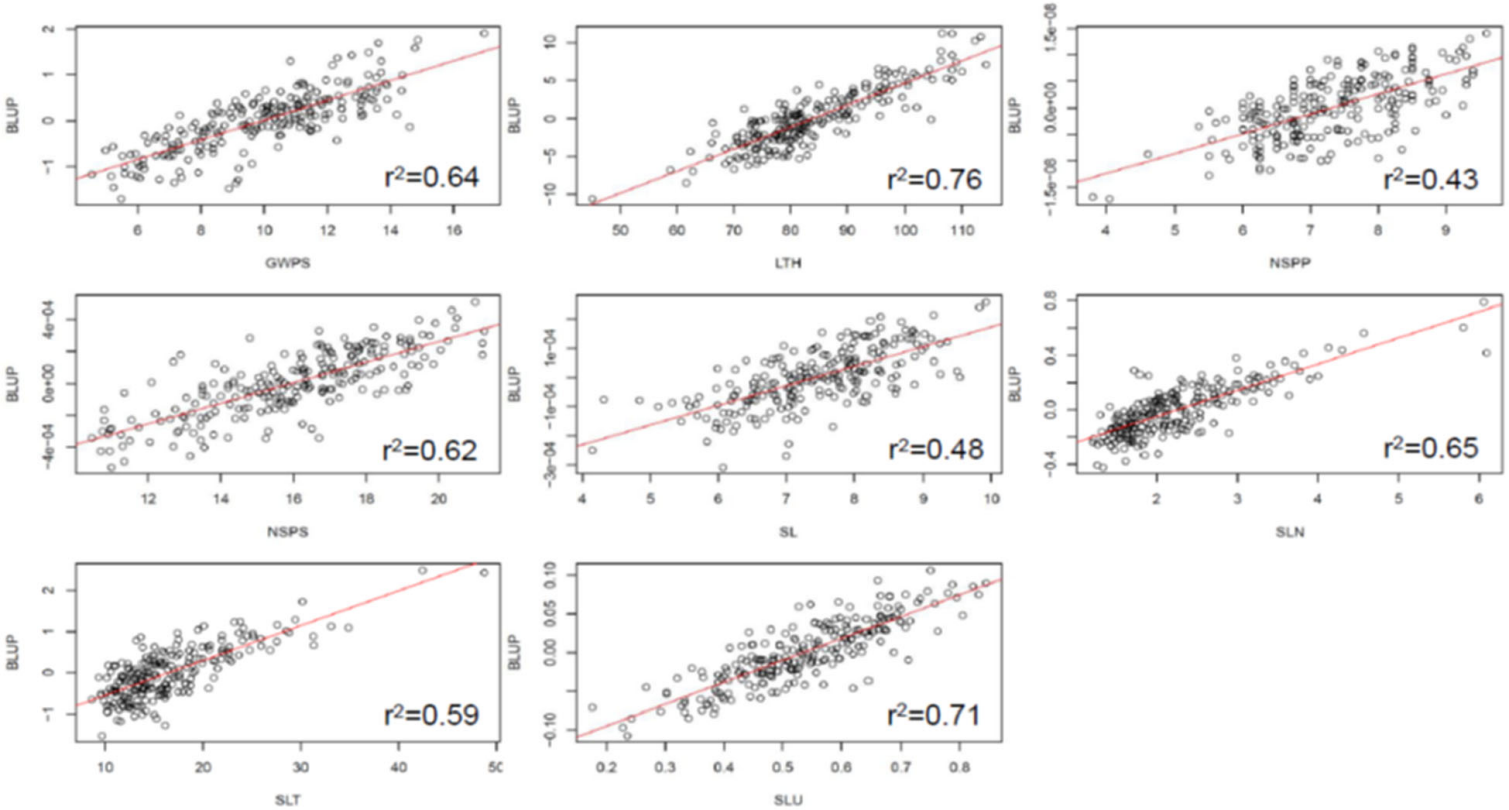
Genomic predictions for the studied traits

To ascertain whether spike-related traits could be predicted using markers, we performed genomic selection (GS) on these traits. Across the eight measured traits, moderate to high prediction values were observed, ranging from r^2^ = 0.43 to 0.76. The lowest and highest predictions were observed for the NSPP and LTH traits, respectively (Fig. 4).

### Identification of candidate genes (CGs)

In total, 279 high-confidence CGs were identified using 48 significant non-redundant SNPs (out of 94). However, no hit was found from the remaining 46 SNPs. To identify functions, CGs were annotated using gene ontology (GO) based on IWGSC RefSeq v1.0. These CGs encode a variety of proteins that include genes involved in accelerating developmental stages such as embryogenesis, lateral root development, vascular differentiation, flower development, cell division, and elongation and differentiation in wheat and related species (Additional file 2; Table S3). Furthermore, based on a survey of published literature on wheat’s life cycle considering spike-related traits, 17 CGs were selected and presented (Table 7) [40–78]. The remaining 262 CGs were not associated with the traits considered in the present study. However, these 262 CGs were associated with several other related traits and thus cannot be ignored (Additional file 2; Table S3).

**Table 7:** List of important candidate genes and their function

Ch.), Trait (E)	Gene Id	Protein domain	Description
SNP_109 (5A); GWPS (E2 & C)	TraesCS5A02G372900	Chaperone DnaJ	DnaJ is a protein involved in the regulation of different defense processes especially in heat stress in the plant. Several previous studies also suggested that this protein family, involved in defense mechanism role during high temperature stress especially during grain filling [40].
	TraesCS5A02G376800	F-box domain	Several proteins are required for growth and development in wheat such as F-box protein, this protein regulates phytohormone abscisic acid (ABA) hormone, this hormone regulates other processes like lateral root development, responses to several stresses, as well as embryo formation, seed dormancy, germination, seedling growth, [41–44].
	TraesCS5A02G378300	AUX/IAA protein	Phytohormones are a vital part of the developmental process and provide signals to regulate this process in a sequenced manner. The auxin played important role in several processes (i) embryogenesis, (ii) lateral root development, (iii) vascular differentiation, (iv) apical dominance, (v) tropic responses, (vi) flower development, (vii) cell division, elongation, and differentiation [45–47].
	TraesCS5A02G381100	Cytochrome P450	Cytochrome P450 proteins involved in several developmental events and enhances resistance to FHB. FHB is a fungal disease of different crops including wheat grown in humid and warm regions globally [48]. It infects wheat heads during flowering and affects the seed development process [48–51].
SNP_218 (4B); SLU (C)	TraesCS4B02G272400	ABC transporter-like	The ABC transporter played important role in grain growth and development and also a defense mechanism to mycotoxin tolerance in wheat. Mycotoxin causes premature bleaching of wheat spikelets, and thus decreases grain yield [52, 53].
SNP_1140 (3B); SLN (C)	TraesCS3B02G309800	AP2/ERF domain	APETALA2/Ethylene-responsive factor (AP2/ERF) played important role in defense mechanisms like abiotic stresses [54] as well as regulating plant growth and development [55].
SNP_2796 (4A); SLU (C)	TraesCS4A02G446700	Sucrose synthase	Sucrose Synthase (SUS) catalyzes the conversion of sucrose to starch [56, 57]. Starch is the major carbohydrate and determines factor for both yield and overall quality of grain [58, 59].
SNP_4595 (7B); NSPP (E2 & C)	TraesCS7B02G095400	Carbohydrate/purine kinase, PfkB, conserved site	Carbohydrate/purine kinase, PfkB, conserved site, kinase enzymes are involved in reversible protein phosphorylation to control different processes such as cellular functions, responses to hormonal, pathogenic, and environmental stimuli, and control of metabolism [60]. The carbohydrate kinases utilize a mutual strategy to determine the reaction between the sugar hydroxyl and the donor phosphate. However, several carbohydrate kinases are allosterically regulated using different strategies, for controlling carbohydrate metabolism [61, 62].
SNP_5026 (6A); NSPS (C)	TraesCS6A02G382400	SWEET sugar transporter	SWEET gene products played diverse roles in essential developmental and physiological processes like growth, senescence and flower, seed endosperm, and pollen development, and regulate critical steps in grain filling, which largely controls the crop yield [63, 64].
SNP_6196 (4A); SLU (C)	TraesCS4A02G157400	Cellulose synthase	Cellulose synthase played important role in cell wall biogenesis and regulation of plant phenotype [65–67].
	TraesCS4A02G097900	Heat shock protein 70 family	Heat shock proteins (HSPs) have a significant role in protein folding and heat-tolerant crops. Wheat is severely affected by heat stress, mainly during the grain filling stage [40].
	TraesCS4A02G315300	MADF domain	MADF protein is involved in the regulation of the phenotype of plants [68].
	TraesCS4A02G058900	MADS-box	MADS-box proteins play important role in reproductive part development regulation; (i) inflorescence architecture, (ii) flowering time control, (iii) floral organ identity determination, and (iv) seed development [69, 70].
SNP_9866 (3B); SLU (C)	TraesCS3B02G367900	Late embryogenesis abundant protein, LEA_2 subgroup	Late embryogenesis abundant (LEA) proteins are involved in the responses and adaptation of plants to several abiotic stresses; (i) dehydration, (ii) salinity, (iii) high temperature, and (iv) cold in wheat [71].
	TraesCS3B02G392600	Transcription factor GRAS	GRAS proteins are involved in the basic metabolic process such as photosynthesis, plant growth, senescence and provide a defense mechanism to photo-oxidative stress [72, 73].
SNP_11184 (1B); SLN, SLT and SLU (E2 & C)	TraesCS1B02G336900	CLAVATA3/ESR (CLE)-related protein 25/26	CLAVATA3 is involved in regulating cell proliferation and differentiation in plant shoots, roots, vasculature, etc. [74].
SNP_13218 (6A); SLU (C)	TraesCS6A02G334200	Alpha-amylase	Alpha-amylase is responsible for starch degradation in cereal crops including wheat during grain germination [75]. Premature production of α-amylase during grain development is considered a quality defect by the wheat industry. Alpha-amylase is also important for the baking industry to improve dough properties and end-product quality [76–78].

**Fig. 5:**
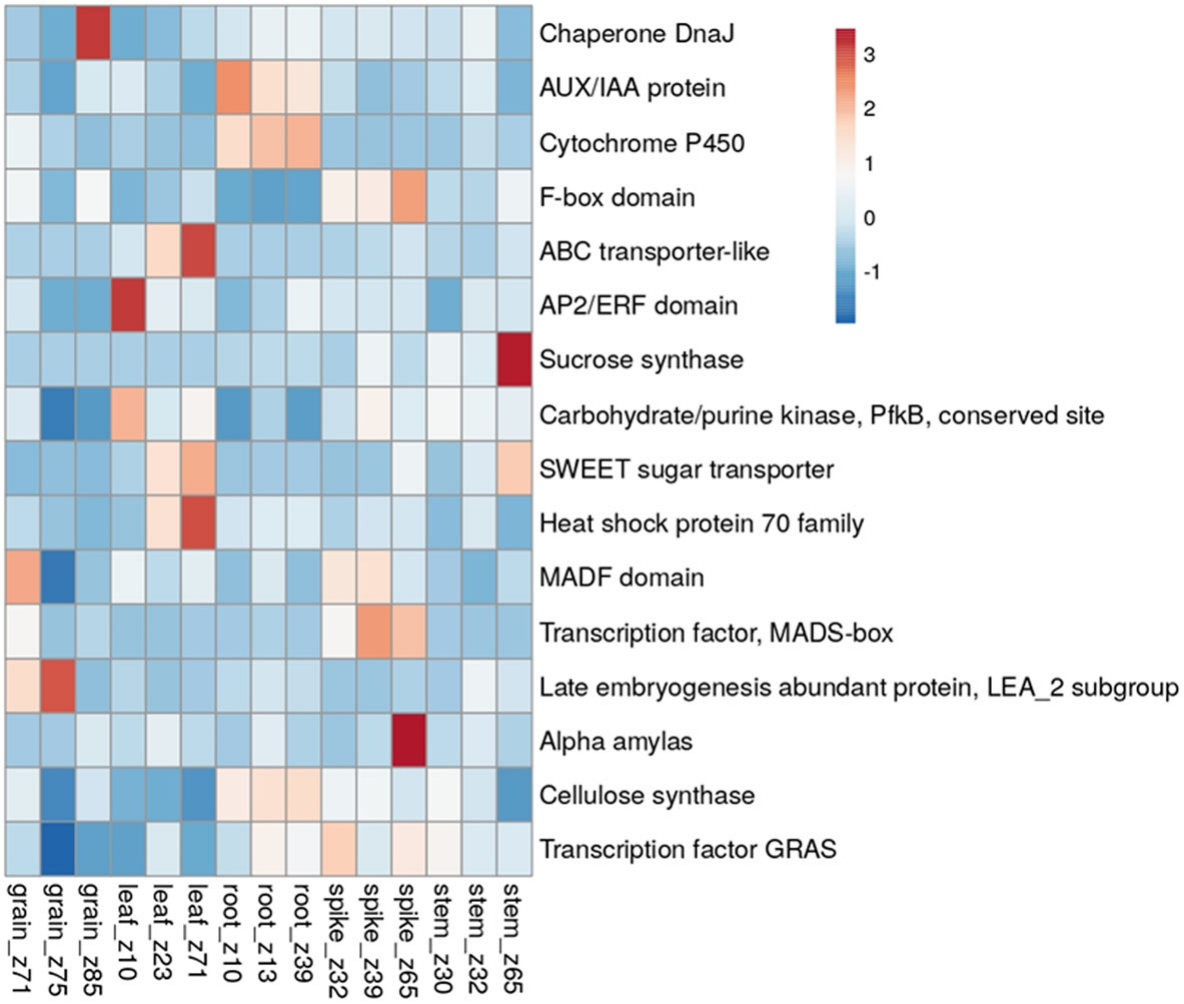
Heat map of in silico gene expression analysis for spike trait candidate genes identified through RNA-seq expression data from wheat expression browser (http://www.wheat-expression.com/)

We further explored the expression analysis of these genes from public repositories. The results of the in-silico gene expression analysis of these CGs are shown in Fig. 5, with related details provided in Table 7. Given that the traits we analysed in the present study are directly related to plant development, differential gene expression data could be used to identify candidate genes. Among the highly expressed differentially expressed genes, TraesCS4A02G446700, which encodes sucrose synthase and is associated with SLU, showed the highest expression in the stem. TraesCS6A02G334200, which encodes alpha-amylase and is associated with SLU, showed high expression in the spike. CGs TraesCS4B02G272400 and TraesCS4A02G097900, which encode ABC transporter-like and heat shock proteins, respectively, were associated with SLU and showed the highest expression in leaves. TraesCS3B02G309800, which encodes AP2/ERF, was associated with SLN and showed the highest expression in leaves. TraesCS5A02G372900, which encodes Chaperone DnaJ and is associated with GWPS, showed the highest expression in grain. The expression pattern of TraesCS1B02G336900 could not be checked, as the corresponding information was not available in the expression database. This CG was associated with SLN, SLT and SLU and encoded CLAVATA3/ESR (CLE)-related protein 25/26, which is known to be involved in the regulation of cell proliferation and differentiation of plant shoots, roots, vasculature, and more. (Table 7).

## Discussion

A major impetus in increasing grain yield comes from finding novel ways of improving crop yields. Improvement in spike-related traits is an important research area that has a direct consequence on grain yield improvements. As screening large segregating populations for these traits in a breeding program can be labour intensive and cost-ineffective, improvements through marker-assisted selection (MAS) or genomic selection is a good option that needs prior marker information or preliminary studies [79, 80]. Thus, understanding the genetics of yield trait components, such as spike morphology, holds the key to wheat yield improvement. In this regard, the availability of wheat genome sequences and subsequently the availability of numerous wheat SNP platforms play important roles in enabling the effective development of cultivars by using genetic resources and genome-enabled breeding.

In wheat, most genome-wide association studies for agronomic and grain yield traits are based on single locus single trait (SLST) analysis [28]. However, it is possible that SLST analysis cannot detect all MTAs [81]. To overcome this limitation, the advanced multi-locus GWAS approach “FarmCPU” (fixed and random model circulating probability unification) has been developed [81]. Hence, in this study, we used FarmCPU, which holds more statistical power and better computational efficiency than other available methods [81] for GWAS analysis, such as EMMA (efficient mixed-model association) [82], EMMAX (EMMA eXpedited) [83] and GEMMA (genome-wide efficient mixed-model association) [84]. FarmCPU eliminates confounding problems arising due to population structure, kinship and multiple testing correction [81, 85–89]. It uses both a fixed-effect model (FEM), a test marker using pseudoQTNs as covariates and a random effect model (REM); tests estimate pseudoquantitative trait nucleotides (QTNs) iteratively [81, 90]. In the FarmCPU model, population structure (PCA) is a fixed effect, kinship is a random effect [81, 88] and model overfitting is avoided by estimating kinship [89]. Given that population structure is well controlled, we did not detect many MTAs for the studied traits. Additionally, Q-Q plots confirmed the suitability of the multi-locus association model used in this study (Fig. 3 and Additional file 1 Fig. S6).

The PCA results obtained in this study suggest that accessions from Mexico harbour greater diversity when compared to India and China, indicating that wheat germplasms from India and China are less diverse. These results are not surprising when considering the large breeding efforts of CIMMYT to diversify their germplasm.

In the present study, we found a total of 136 MTAs for all eight traits in the individual environment and combined data. These MTAs were distributed over 18 wheat chromosomes. Interestingly, a large number of MTAs were from subgenome A, followed by B, and the least was from subgenome D. Since wheat polyploidization involves historical hybridization of the A, B and D genomes, fewer MTAs from the D-genome indicate a founder effect of the wheat population. In the present study, we observed wide ranges in the values of measured traits, as indicative of their distribution (Additional file 2; Table S1). ANOVA of the phenotypic data for different spike-related traits indicated significant genotypic (G) and G X E interactions for most of the studied traits. More MTAs were found in E2 than E1, which indicates the environmental influence of the studied traits. These environmentally specific MTAs may facilitate breeding for specific environmental conditions [91].

Environmental (meteorological) data from both years exhibited large differences in mean temperature and rainfall, suggesting that uneven distribution of these two parameters (over the crop growing period) can lead to large differences in wheat traits (Additional file 1; Table S2). Hence, variation was significantly different in both environments and supports the consideration of two years as two environments. Interestingly, most promising traits, such as GWPS, NSPS, NSPP, SL, LTH and SLU, displayed high heritability and were consequently less influenced by environmental changes, indicating the robustness of these traits. Nevertheless, we measured traits that display strong environmental influence as spike-related traits could be influenced by many environmental variables that make routine wheat breeding difficult. Similar to the present study, genotype-by-environment (G X E) interactions have also been reported previously for traits such as spikelet per spike, spike length, the number of spikes per plant, etc. [12, 92–94]. Zhao et al. [12] also observed high heritability (more than 50%) for NSPP, SL, NSPS, GWPS, LTH and SLU, whereas two traits, SLT and SLN, showed heritability of less than 50% (Table 2). Present and previous research suggests the complexity of the SLURTs as they often interact with the environment, demonstrating the difficulty in genetic improvement of SLU by direct selection through conventional wheat breeding programs. Therefore, it is important to perform a genetic analysis of SLURTs in wheat using molecular breeding programs.

In wheat, previous studies [95, 96] have found that plants with bumpy spike layers have a high yield potential. Therefore, uniform spikes (even spike layers) distributed in limited packed horizontal space may reduce photosynthetic rates and yield in wheat. In contrast, uneven spike-layer distribution exposes large leaf surface areas to sunlight causing higher photosynthesis which in turn results in higher wheat yield. Thus, plants with suitable uneven spike layers may be an ideotype for higher yield in wheat [12]. Negative correlations between yield and SLU observed in the present study further support this view. A negative correlation between yield and SLU was also observed in a biparental mapping study [12]. Similarly, Yao [95] and Hu [96] showed that SLU had a significantly negative correlation with yield potential in wheat, and plants with a slight difference in spike heights among tillers (uneven spike-layer to a certain extent) resulted in higher yield potentials. Grain per spike is a primary component of wheat yield, and spikelet number is a primary factor affecting the grains per spike in wheat. The photoperiod and temperature are the two major environmental factors that control spikelet and floret primordia initiation in wheat [17, 97–100]. Similarly, different developmental stages of spikes affect spikelet number and grain yield [3, 101, 102]. Additionally, spike length variation also affects grain per spike and thus plays an important role in improving wheat yield [14]. We found an overlap of six MTAs for three traits with earlier studies. Six MTAs for three traits were also found within the reported QTL interval for grain yield (Additional file 1 Fig. S7). Moreover, in the present study, four stable MTAs were found (Table 4). Three MTAs were for LTH and one MTA was for SLU. Out of three MTAs for LTH, SNP_2448 was mapped within the reported QTL interval for tiller number [103] on chromosome 1A (Additional file 1 Fig. S7). In addition, six multi-trait MTAs were also detected (Table 5). These findings will provide useful information for wheat breeding programs.

Several significant correlation combinations were observed in this study, such as LTH positively correlated with SLN and SLT in E1 and the combined; similarly, LTH was negatively correlated with SLU in E1 and the combined analysis. Likewise, SLU was negatively correlated with SLN and SLT in both the environments and in the combined analysis. Conversely, a positive correlation between LTH and SLU was found in rice (*Oryza sativa*) [11], indicating that the genetic mechanisms controlling SLU in wheat and rice are likely under the control of diverse mechanisms. Furthermore, SLN was positively correlated with SLT in all the data sets (Additional file 1 Fig. S2, S3 & S4), a similar pattern of correlation combination was reported by [12] in wheat.

As illustrated in Fig. S1 (left panel) (Additional File 1: Fig. S1), SLN and SLU were identical (value =1) when the genotype within a plot exhibited uniform heights. However, when the spikes are of different heights (right panel of Fig. S1) the SLN and SLU values are not identical. Thus, the value of SLU is 0.5, but the value of SLN is 2. Hence, SL = SLT, SLU = 1, SLN = 1 when all spikes are of identical heights (identical SL). Nevertheless, in this study, we did not detect identical trait values for these traits, a negative or positive correlation occurred between the three derived traits. As both SLU and SLN are inverse of each other, strong negative correlations, − 0.85***, − 0.84*** and − 0.87***, between SLU and SLN were observed for E1, E2 and the combined, respectively (Additional File 1: Fig. S2, S3, S4).

Thus, spike height (spike length or SL) plays an important role in the SLT calculation. Therefore, if all spikes are of the same height, then SLT = SL. However, if spike height (SL) increases or decreases, SLT also correspondingly increases or decreases, respectively. Positive correlations were observed between SL and SLT, 0.24***, 0.21*** and 0.23***, for E1, E2 and combined, respectively. (Additional File 1: Figs. S2, S3, S4). Similarly, SLT increases or decreases depending on the increase or decrease in SLN (as SLN=SLT/SL) and strong positive correlations were observed between SLN and SLT, 0.94***, 0.88*** and 0.94***, for E1, E2 and combined, respectively (Additional File 1: Fig. S2, S3, S4). Likewise, SLU is inversely related to SLT (SLU=SL/SLT); hence, strong negative correlations, − 0.81***, − 0.76*** and − 0.83***, between SLU and SLT were observed for E1, E2 and the combination of E1 and E2, respectively (Additional File 1: Figs. S2, S3, S4). Hence, calculated traits such as SLN and SLU display strong concordance as several significant MTAs overlapped (Table 5).

In this study, after applying FDR correction, only five MTAs were retained (Table 6). It is well known that multiple test correction steps (such as FDR) in addition to reducing the number of undesirable false-positive associations can lead to the loss or disappearance of true associations (false negatives) [104]. Hence, MTAs that disappear after the application of FDR correction should not be ignored entirely. Mainly when MTAs reported within or near previously reported QTL intervals.

It is difficult to know coinciding hits (with previous studies) mainly due to the usage of different mapping populations, population structure, and LD decay. In this study, a conservative threshold of 50 Mbp was used to locate overlapping QTLs. More research is needed to better understand these genomic regions by identifying and characterizing the candidate genes. The present study detected MTAs within or near previously reported QTL intervals, ensuring the robustness of the analysis. It also suggests the possible usage of these potential MTAs in molecular breeding. Large numbers of MTAs identified in the present study were also subjected to further scrutiny to identify the most important MTAs which could be recommended for marker-assisted recurrent selection (MARS) or marker-assisted selection (MAS). MTAs that fulfilled at least one or more of the following criteria were selected for this purpose: (i) lowest *P-*value, (ii) qualified FDR multiple correction, (iii) common among two or more traits, (iv) stable (identified in both the environment and combined data) and (v) detected in earlier studies (including both interval mapping and GWAS). A total of 16 MTAs were found using the above criteria for different traits (Table 8). One such MTA is SNP_10924. This MTA was associated with SLU, present in both E1 and E2 and the combined environments and surpassed the FDR threshold. Furthermore, the MTAs observed in this study could be grouped into QTL regions considering chromosome-wise linkage disequilibrium (LD). Based on this, five groups were identified involving 14 MTAs (12 SNPs) for three different environments based on LD decay distance on particular chromosomes (Table 9). The distance among the remaining MTAs was greater than the LD decay distance for a particular chromosome. The following MTA groups were observed. (i) In E1, a single group was observed of two SNPs (SNP_1870 and SNP_10789) mapped on the 5A chromosome and associated with SLU. (ii) In E2, two groups were observed. The first group consisted of two SNPs (SNP_4799 and SNP_3408) on chromosome 5B and was associated with SLN and SLU, the second group consisted of four SNPs (SNP_5327, SNP_10576, SNP_8090 and SNP_5909) mapped on the 6D chromosome and associated with SLU. (iii) In the combined data (E1 and E2), two groups were also observed, the first group consisted of two SNPs (SNP_1852 and SNP_2933) mapped on the 6A chromosome and associated with LTH and the second group consisted of two SNPs (SNP_4799 and SNP_3408) mapped on chromosome 5B and associated with SLT. Two SNPs (SNP_4799 and SNP_3408) were observed both for E2 and the combined data.

**Table 8:** Summary of most important MTAs for MARS or MAS

SNP	Allele.	Chr.	Pos.	Description
SNP_11184	T/C	1B	277	Associated with more than one trait such as SLN; E2 & C, SLT; E2 & C, SLU; E2 & C and also qualified FDR for SLN
SNP_13306	C/T	6B	51	Associated with more than one trait such as SLN; E2, SLT; E2, SLU; C
SNP_1870	T/C	5A	27	Associated with more than one trait such as SLT and SLU; E1
SNP_3408	A/G	5B	106	Associated with more than one trait such as SLN; E2, SLU; E2, SLT; C
SNP_4799	C/G	5B	106	Associated with more than one trait such as SLN; E2 & C, SLU; E2, SLT; C and also qualified FDR for SLN
SNP_5177	G/A	2D	25	Associated with more than one trait such as GWPS; E1 & C, SLU; E1 & E2, SL; C, SLN; C
SNP_1800	C/A	6A	159	Associated with LTH and found in both the environments and combined data
SNP_1852	A/C	6A	175	
SNP_2448	G/A	1A	127	Associated with LTH and found in both the environments, combined data and also reported in the earlier study [103]
SNP_10924	A/G	5B	102	Associated with SLU, qualified FDR, and found in both the environments and combined data.
SNP_6294	C/T	6A	33	Associated with NSPP and qualified FDR
SNP_109	C/T	5A	169	Associated with GWPS and reported in the earlier study [105]
SNP_7209	C/G	4A	177	Associated with GWPS and reported in the earlier study [106]
SNP_12067	T/C	2A	132	Associated with LTH and reported in the earlier study [107]
SNP_598	C/A	2A	16	Associated with NSPP and reported in the earlier study [108]
SNP_454	A/G	4B	64	

Chr: chromosome, Pos: position in cM

**Table 9:** Details of MTAs groups (QTLs) observed on the basis of chromosome-wise LD decay distance

Trait	SNP groups (QTL region)	Chr	Pos.	LD
SLU; E1	SNP_1870	5A	27	3.0
	SNP_10789	5A	29	
SLN and SLU; E2	SNP_4799	5B	106	2.5
	SNP_3408	5B	106	
SLU; E2	SNP_5327	6D	186	4.5
	SNP_10576	6D	187	
	SNP_8090	6D	187	
	SNP_5909	6D	185	
LTH; C	SNP_1852	6A	175	3.5
	SNP_2933	6A	175	
SLT; C	SNP_4799	5B	106	2.5
	SNP_3408	5B	106	

E1: Environment 1, E2: Environment2 and C: Combined data (E1 and E2), Chr: chromosome, Pos: position in cM, LD: LD decay distance

Given that the traits described in this study are environmentally sensitive, we still obtained high genomic prediction values of most of the traits based on the markers. This suggests the merits of genomic selection in breeding for SLURT traits and could be implemented for breeding when labour-intensive phenotyping is not feasible.

An effort was also made to identify CGs using high confidence MTAs, resulting in the identification of a total of 279 CGs, suggesting their possible involvement in different biological processes (Additional file 2; Table S3). Important putative CGs that played important roles in different biological pathways in wheat were selected (Table 7). Domains such as MADS boxes are known to play important roles in reproductive development, regulation, inflorescence architecture, flowering time control, floral organ identity determination and seed development [69, 70]. Likewise, the transcription factor GRAS was also detected, which is well known for its involvement in basic metabolic processes such as photosynthesis, plant growth, senescence and more [72, 73].

## Conclusions

The present study is the first report on MTAs for SLURTs in multiple environments using multi-locus GWAS. This study provides insights into the genomic regions of SLURTs. The MTAs identified herein may facilitate breeding new wheat varieties with logically applied spike-layer distribution through MAS. The identified MTAs will be useful for MAS after further validation through suitable mapping populations segregating for these significant MTAs. For high-throughput SNP genotyping, a suitable KASP (Kompetitive Allele-Specific PCR) assay may also be developed using SNP tags of desirable alleles identified through GWAS. CGs identified in the present study should be further validated through functional genomics approaches and may be utilized for developing CG-based markers for their further utilization in CG-based association mapping. In conclusion, we suggest that early-stage selection during breeding programs may focus on the aspect of the suitable vertical spatial distribution of spikes to target higher yield potential.

## Methods

### Wheat association mapping panel and SNP genotyping

The association mapping panel used consisted of 225 diverse wheat genotypes (Additional file 1; Table S1), which is a subset of the original spring wheat reference set (SWRS) of 330 wheat genotypes [109]. Seed material was obtained from the International Maize and Wheat Improvement Centre (CIMMYT), Mexico [109]. Genotyping was performed by CIMMYT through outsourcing using diversity array technology (DArT) in combination with next-generation sequencing platforms known as DArT-seq [109]. Out of the 17,937 SNP markers made available for the original set of 330 genotypes, 9627 (53.67%) were mapped on the genetic map. A total of 5582 markers were finally retained after further filtering the 9627 genetically mapped markers for a minor allele frequency of 5% and missingness (30%).

### Field experiment

The association panel of 225 diverse spring wheat genotypes was raised in a simple lattice design with two replications for two years (2017–18 and 2018–19) at the Agriculture Research Farm, Ch. Charan Singh University, Meerut, UP, India; 28.984644°N and 77.705956°E.

Lattice designs are very useful incomplete block designs for plant breeders, as these designs have more flexibility in choosing the number of replications depending upon the availability of resources [110]. The lattice design can be a simple lattice if the design has two replications of the treatments [110]. The replication number and the treatments are flexible in a simple lattice design and thus useful for testing a large number of treatments [111]. Several recent GWAS in crops, including wheat and rice, have used the alpha lattice design with two replications [112, 113].

In this study, each year was treated as one environment, thus making two environments (E1, E2). Data from both years were significantly different for most of the environmental parameters after applying a t-test on both year’s meteorological data (Additional file 1; Table S2). Each genotype was represented by a plot of 3 rows of 1.5 m each, with a row-to-row distance of 0.25 m. The total number of blocks were 15, with each block containing 45 rows, i.e., three rows of each genotype. Five plants per row were used for data recording. First, second, third, fourth and fifth irrigations were conducted 21, 45, 60, 90 and 100 days after sowing. Standard field management practices (i.e., 200 kg/ha fertilizer; N:P:K = 8:8:8) were followed for both environments.

### Phenotyping

The data on each of the 225 genotypes were recorded for the spike traits according to the following procedure: Number of spikes per plant (NSPP); total number of normal spikes were counted from five randomly selected plants of each plot, out of which five randomly selected spikes were used to calculate other different parameters such as (a) the number of spikelets per spike (NSPS) (spikelet number was counted from the basal sterile spikelet to the top fertile spikelet), (b) spike length (SL) (SL was measured from the base of the spike to the tip, excluding awns), (c) grain weight per spike (GWPS) (after threshing and cleaning of spikes, the grain weight was calculated in grams). Finally, the mean value over the five spikes of each genotype was considered as a final trait value. The lowest tiller height in cm (LTH) was measured from ground level to the tip of the spike (excluding awns), plant height (PH) was measured from ground level to the tip of the spike (excluding awns), and LTH and PH were calculated from a plant selected for NSPP. Similarly, spike-layer thickness (SLT) was calculated from the three traits viz.; PH, LTH and SL (SLT = PH–LTH + SL), spike-layer number (SLN) was calculated as SLN=SLT/SL, and spike-layer uniformity (SLU) was calculated as SLU=SL/SLT. The biological meaning of these traits is presented in Table 1. Measurements for SLURTs were done following [12].

To ascertain the relationship between the spike traits and grain yield per plot (GYPP; in grams), average grain yield data was also measured (size of plots) for two years (2017–18 and 2018–19). The total grain yield of a per m^2^ plot was weighed in grams. Since the data were considered in another unpublished study on the yield traits, only pairwise Pearson correlation analysis was presented.

### Statistical analysis

Descriptive statistics of the measured phenotypes, including the mean, range, standard error and coefficients of variation (CV), were estimated using SPSS Inc. 2008 [114]**.** Replicated data were used to calculate ANOVA and heritability in a particular year/environment. The mean data of both replications of the individual year/environment of each trait were used to conduct GWAS and correlation analyses. (Additional file 2; Table S2). For the combined analysis over the two environments, least-square means were calculated in the R package *“Emmeans”* version 1.4.8 (https://cran.r-project.org/web/packages/emmeans/index.html). Pairwise correlation analysis (Pearson’s method) was performed in the R package *Performance Analytics* among eight traits as well as grain yield. Likewise, the *Agricolae* R package was used for the estimation of ANOVA using the additive main effects and multiplicative interactions (AMMI) model. Broad sense heritability (*H*^*2*^) was calculated according to [115] from the genotypic variance (σ^2^g) and phenotypic variance (σ^2^p) using Microsoft Excel 2010. F-values were calculated for each trait using the environment and replication varieties that were used for the combined analysis of the traits.

### Population structure and linkage disequilibrium (LD) analyses

To understand the population structure, PCA was performed from a set of polymorphic SNPs implemented in TASSEL version 5.0 and displayed using [116]. To compute pairwise LD between the markers, TASSEL version 5.2.54 was used. Chromosome-wise, as well as whole-genome LD decay, was conducted in TASSEL 5 from a set of genetically anchored markers, and decay distance (in cM) was plotted. The LD decay of the panel was reported in detail in our previous publication [30].

### Marker trait association (MTA) analysis

GWAS was performed using 5582 genetically mapped markers through FarmCPU implemented in GAPIT version 3 [81, 117]. Manhattan and QQ plots were generated using R packages, qqman version 0.1.4 [118]. The *P*-value threshold was set as 0.001 (−log10 [*p*-value] =3.0). QQ plots were used to examine model fitting which accounts for population structure. False discovery rate (FDR) correction criteria were also applied to all the identified MTAs with corrected *p*-values < 0.05 to remove false-positive associations.

### Comparison of MTAs detected to historical QTL regions

The MTAs identified were also compared with previously reported QTLs/MTAs for the same traits and grain yield. The physical positions of all MTAs detected in the present study and previously reported QTLs/MTAs were identified through Ensembl Plants [version 50; https://plants.ensembl.org/Triticum_aestivum/Info/Index]. The results were compared based on the physical position of the particular marker on a particular chromosome for the same trait or grain yield. Representative chromosomal maps were prepared for the comparative presentation of all MTAs detected in the present study and previously reported QTLs/MTAs for each trait on an individual chromosome using Map chart software [119].

### Genomic selection (GS)

The GS method cBLUP implemented in GAPIT version 3 was used to obtain GS values across the measured traits. Graphics of GS were generated in the R statistical environment using the base package and functions (R Core Team, **https://www.rproject.org/contributors.html**).

### Putative candidate gene (CG) identification

A total of 136 significant MTAs were identified in E1, E2 and the combined data, out of which 94 unique MTAs were subjected to the identification of CGs for all the study traits (Additional file 2; Table S3). Candidate genes for these MTAs were identified by aligning the related GBS sequences to wheat genome assembly IWGSC1.0 (IWGSC, 2018), which is hosted on the Ensembl database (http://www.ensembl.org/info/docs/tools/vep/index.html). High-confidence annotated genes were retrieved from a chromosome-wise LD decay distance window for each identified MTA. This window size varies for each MTA depending on the local LD decay for each chromosome. The gene ontology (GO) annotation information of these candidate genes (CGs) was extracted from the IWGSC website (http://www.wheatgenome.org/). In silico gene expression analysis was also conducted to identify CGs using RNA Seq expression data from Wheat Expression Browser (http://www.wheat-expression.com/). A heatmap was generated for the presentation of expression data of genes in different stages and tissues considering the selected domain relevant to the studied traits.

## Supplementary Information

**Additional file 1: Table S1:** List of 225 spring wheat reference set genotypes. **Table S2:** Comparative analysis of environmental factors in two different environments/years using t-test. **Fig. S1.** The biological meaning of spike layer uniformity (the consistency of the spike distribution in the vertical space) related traits (SLURTs) shown with wheat plants having similar tillers height (a) and different tiller height (b). In (a) only one spike layer can be seen from the vertical perspective (SLN = 1) as spikes per plant have consistent vertical distribution (SLU = 1) and the SLT was identical to one spike length (SL) whereas in (b) two spike layer can be seen from the vertical perspective (SLN = 2) as spikes per plant have inconsistent vertical distribution (SLU = 0.5) and the SLT was identical to two spike length. A detailed description of SLURTs is provided in Table 1. Note: In the figure, immature wheat plants are shown just to illustrate the biological meaning of SLURTs. However, data for SLURTs were recorded on mature plants. In the figure scaling is approximate. The figure is based on the publication by Zhao et al. [12]. **Fig. S2.** Pairwise Pearson correlation among the eight SLURTs in E1 and grain yield (GYPP). A single asterisk (*) represents 0.05 level of significance. A double asterisk (**) represents 0.01 level of significance and a triple asterisk (***) represents 0.001 level of significance. **Fig. S3.** Pairwise Pearson correlation among the eight SLURTs in E2 and grain yield (GYPP). A single asterisk (*) represents 0.05 level of significance. A double asterisk (**) represents 0.01 level of significance and a triple asterisk (***) represents 0.001 level of significance. **Fig. S4.** Pairwise Pearson correlation among the eight SLURTs and grain yield (GYPP) for the combined E1 and E2 environments. A single asterisk (*) represents 0.05 level of significance. A double asterisk (**) represents 0.01 level of significance and a triple asterisk (***) represents 0.001 level of significance. **Fig. S5.** Manhattan plots obtained after using FarmCPU for eight SLURTs (a) GWPS (b) LTH (c) NSPP (d) NSPS (e) SL (f) SLN (g) SLT (h) SLU under E1 (above numbers) and E2 (below numbers) conditions. Numbers correspond to wheat chromosome. **Fig. S6.** QQ-plots to visualize the deviation of observed *p* values from expected *p* values (based on null hypothesis) for eight SLURTs (a) GWPS (b) LTH (c) NSPP (d) NSPS (e) SL (f) SLN (g) SLT (h) SLU under E1 and E2 conditions. **Fig. S7 (i-v).** Comparison of MTAs or MTA groups, detected in the present study, with historical QTLs/MTAs in different chromosomes; MTAs detected in known flanking regions or locations of QTLs/MTAs for same traits or grain yield are depicted in green color and MTAs found near to flanking regions or locations (less than 50 Mb) of QTLs/MTAs depicted in red colour. Flanking markers are shown with the same colour and with the reference details of the previous study. Novel MTAs detected in the present study are presented without any colour. The corresponding physical distances (Mb) of the QTL/MTA regions on each chromosome were obtained by blasting the flanking sequences of markers, depicted on the right side of each figure, to the Chinese Spring RefSeq v1.0. GY; grain yield, TN; tiller number.

**Additional file 2: Table S1.** Descriptive statistics details for all the studied traits. **Table S2.** Phenotypic data of environment 1 (E1: 2017–18), environment 2 (E2: 2018–19) and mean of E1 and E2. **Table S3.** List of candidate genes identified using MTAs detected in E1, E2 and combined (E1 and E2).

## Data Availability

The phenotypic datasets created and analysed during the current investigation for the GWAS are accessible in supplemental table (Additional file 2; Table S2**)**. The genotypic dataset used in this work is accessible from the corresponding author upon reasonable request for non-commercial purposes. As the dataset is subset of the larger panel of accessions from CIMMYT that are genotyped, the corresponding genotypic information can also be obtained from the Nature communication paper [109]; DOI: 10.1038/s41467-020-18404-w).

## Abbreviations

SLURT: Spike-layer uniformity related trait
GWAS: Genome-wide association study
AM: Association mapping
LD: linkage disequilibrium
NSPP: Number of spikes per plant
SL: Spike length
NSPS: Number of spikelets per spike
GWPS: Grain weight per spike
LTH: Lowest tiller height
SLT: Spike-layer thickness
SLN: Spike- layer number
SLU: Spike-layer uniformity
PH: Plant height
GYPP: Grain yield per plot
SNPP: Spike number per plant
FSN: Fertile spikelet number
SN: Spikelet number
SSN: Sterile spikelet number
GNPS: Grain number per spike
MTA: Marker trait association
SLST: Single locus single trait
CG: Candidate gene
SNP: Single nucleotide polymorphism
GS: Genomic selection
QTL: Quantitative trait loci
DArT: Diversity arrays technology
RIL: Recombinant inbred line
MAS: Marker-assisted selection
FDR: False discovery rate
FarmCPU: Fixed and random model Circulating Probability Unification.
MARS: Marker-assisted recurrent selection
MABC: Marker-assisted backcrossing
