# Supplementary material for: Multi-locus genome-wide association mapping for spike-related traits in bread wheat (Triticum aestivum L.)
Source: BMC Genomics. 2021 Aug 5;22:597. doi: 10.1186/s12864-021-07834-5 (PMC8340506; doi:10.1186/s12864-021-07834-5)

**Additional file 1; Table S1:** List of 225 spring wheat reference set genotypes

| S. No. | Name | S. No. | Name |
| --- | --- | --- | --- |
| 1 | ZAMBESI | 71 | II12107-3R-6M-2R |
| 2 | PERRERSON ML68-4 | 72 | Citr 14998 |
| 3 | V18 | 73 | Citr 15308 |
| 4 | GRAY JD253 | 74 | RUPERTJ CAR 1035 (27035) |
| 5 | GRAY JD416 | 75 | KING-HING-1 |
| 6 | GRAY JD629 | 76 | AKBUGDAY |
| 7 | GRAY JD738 | 77 | AKBASAK |
| 8 | GRAY JD 757 | 78 | ZERDAKIA |
| 9 | GRAY JD 893 | 79 | S-1 |
| 10 | GRAY JD930 | 80 | VIJAYA |
| 11 | GRAY JD1024 | 81 | BCH/HUAC |
| 12 | GRAY JD1032 | 82 | PVN/ /CAR422/ANA/5/BOW/CROW/ /BUC/PVN/3/YR/4/TRAP#1 |
| 13 | GRAY JD1102 | 83 | WEEBILLI |
| 14 | GRAY JD1196 | 84 | WH 542 |
| 15 | GRAY JD1278 | 85 | KAUZ/ /ALTAR 84/AOS/3/KAUZ |
| 16 | GRAY JD1447 | 86 | OTUS |
| 17 | C-40-1 | 87 | SKAUZ82/FCT |
| 18 | MOSKOVSKAYA 21 VIR 48760 | 88 | SIRKKU |
| 19 | W-33-A | 89 | OTUS/TOBA97 |
| 20 | W-36 | 90 | BONASA |
| 21 | W-37 | 91 | KAUZ/ /BOW/NKT |
| 22 | W-39 | 92 | ECIJA:AE |
| 23 | W-63 | 93 | SW89.5181/KAUZ |
| 24 | W-65 | 94 | MINO |
| 25 | W-67 | 95 | CHIBIA/PASTOR/ /CHIBIA |
| 26 | W-79 | 96 | VERDIN |
| 27 | W-83 | 97 | VEE/PJN/ /2*TUI/3/WH576 |
| 28 | W-84 | 98 | MUNIA/3/RUFF/FGO/ /YAV79/4/PASTOR |
| 29 | W-85 | 99 | CNDO/R143/ /ENTE/MEXI_2/2/ AEGILOPS SQUARROSA (TAUS)/4/WEAVER/5/IRENA |
| 30 | W-86 | 100 | PBW343/TONI |
| 31 | W-88 | 101 | MILAN/KAUZ/6/TOB/ERA/ /TOB/CN067/3/PLO/4/VEE#5/5/KAUZ |
| 32 | W-91 | 102 | VEE#8//JUP/BJY/3/F3.71/TRM/4/2*WEAVER/5/HAHN/2*WEAVER/6/WEAVER |
| 33 | BW110 | 103 | CHUM18/7*BCN |
| 34 | QITAI-CHUN-4-ST-119 | 104 | URES/BOW/ /OPATA/3/PASTOR |
| 35 | TURPAN SHAN YUEH H C M | 105 | CHAPIO |
| 36 | TOKSUN SPRING 2 ST-122 | 106 | SITELLA |
| 37 | WANNIAN 2 | 107 | TUI |
| 38 | HONG DUAN MANG | 108 | DUCULA |
| 39 | CItr 2346 | 109 | SCHOMBURGK |
| 40 | YANTAGBAY | 110 | KATUNGA |
| 41 | LOROS | 111 | BARUNGA |
| 42 | CItr 4309 | 112 | KRICHAUFF |
| 43 | CItr 4315 | 113 | WESTONIA |
| 44 | CItr 4901 | 114 | BATAVIA |
| 45 | CItr 5088 | 115 | WW425 |
| 46 | JENKIN | 116 | SITTA |
| 47 | BIHAR-51 | 117 | CETTLA |
| 48 | BIHAR-59 | 118 | TAM200/TUI |
| 49 | BIHAR-66 | 119 | SUPER SERI #1 |
| 50 | ALI-BEN-MAKLOUL | 120 | CROC_1/AE.SQUARROSA (205)/ /KAUZ |
| 51 | WILBUR | 121 | HXL7573/2*BAU |
| 52 | Citr 7030 | 122 | DHARWAR DRY |
| 53 | CANDEAL:AE | 123 | PAVON TALL |
| 54 | Citr 7089 | 124 | KAUZ TALL |
| 55 | Citr 7289 | 125 | PASTOR/BAV92 |
| 56 | MARGARITOVO | 126 | CNDO/R143/ /ENTE/MEXI_2/3/AEGILOPS SQUARROSA(TAUS)/4/ WEAVER/5/PASTOR |
| 57 | REWARD | 127 | BERKUT |
| 58 | DORSETT-PH-3892 | 128 | VOROBEY |
| 59 | Citr 8327 | 129 | ALTAR 84/AE.SQUARROSA (224)/ /2*YACO/3/BAV92 |
| 60 | DORSETT-PH-2140 | 130 | MILAN/KAUR/ /DHARWAR DRY/3/BAV92 |
| 61 | GRANDE-DEL-MONTE | 131 | KABY/BAV92/3/CROC_1/AE.SQUARROSA (224)/ /OPATA |
| 62 | ROJO BARBON | 132 | BJY/COC/ /PRL/BOW/3/MILAN/KAUZ/4/BAV92 |
| 63 | LOVE-HH-129 | 133 | ATTILA/BAV92/ /PASTOR |
| 64 | DORSETT-PH-6955 | 134 | SARATOVSKAYA 29 |
| 65 | DORSETT-PH-6993 | 135 | TSELINNAYA-YUBILEINAYA |
| 66 | DORSETT-PH-7017 | 136 | OMSKAYA-32 |
| 67 | DORSETT-PH-7053 | 137 | KE FENG 2 |
| 68 | DORSETT-PH-6927 | 138 | NEW LONG MAI 19 |
| 69 | DORSETT-PH-7150 | 139 | LONG MAI 23 |
| 70 | LOVE-HH-G23B | 140 | AC BARRIE |

| S. No. | Name | S. No. | Name |
| --- | --- | --- | --- |
| 141 | AC VISTA | 183 | SAN CAYETANO S 97 |
| 142 | CHUM18/SERI | 184 | TANORI F 71 |
| 143 | SABUF/4/ALTAR 84/AE.SQUARROSA (224)/ /YACO/3/CROC_1AE.SQUARROSA (205)/ /F27202 | 185 | TINAMOU I1 |
| 144 | PASTOR/3/KAUZ*2/OPATA/ /KAUZ | 186 | TOBARITO M 97 |
| 145 | CROC_1/AE.SQUARROSA (205)/ /KAUZ/3/SASIA | 187 | V-17 |
| 146 | CROC_1/AE.SQUARROSA (205)/ /KAUZ/3/SASIA | 188 | YAQUI 50 |
| 147 | CROP_1/AE.SQUARROSA (205)/ /BORL95/3/ATTILA | 189 | ZAMINDAR 80 |
| 148 | PASTOR/ /TRAP#1/BOW/3/CHEN/AEGILOPS SQUARROSA (TAUS)/ /BCN | 190 | CNO/7C |
| 149 | FILIN/IRENA/5/CNDO/R143/ /ENTE/MEXI_2/3/AEGILOPS SQUARROSA (TAUS) /4/WEAVER | 191 | FIRETAIL |
| 150 | CHEN/AEGILOPS SQUARROSA (TAUS)/ /BCN/3/BAV92 | 192 | PFAU/VEE#9/ /URES |
| 151 | CHEN/AEGILOPS SQUARROSA (TAUS)/ /BCN/3/CMH81.38/2*KAUZ | 193 | BJY/COC/ /PRL/BOW/3/MILAN/KAUZ/4/BAV92 |
| 152 | CROC_1/AE.SQUARROSA (205) / /KAUZ/3/PASTOR | 194 | CHIL/BOMB |
| 153 | ESDA/ /ALTAR 84/AE.SQUARROSA (211)/3/ESDA/4/CHOIX | 195 | REDWING |
| 154 | CROC_1/AE.SQUARROSA (205)/ /KAUZ/3/ATTILA | 196 | D67.2/P66.270/ /AE.SQUARROSA (320)/3/CUNNINGHAM |
| 155 | FISCAL | 197 | D67.2/P66.270/ /AE.SQUARROSA (320)/3/CUNNINGHAM |
| 156 | CHIBIA/4/PGO/ CROC_1/AE.SQUARROSA (224)/3/2*BORL95 | 198 | PARUS/3/CHEN/AE.SQ/ /2*OPATA |
| 157 | EXCALIBUR | 199 | ATTILA/3*BCN/3/CROC_1/AE.AQUARROSA (224)/ /OPATA |
| 158 | JANZ | 200 | YANAC/3PRL/SARA/ /TSI/VEE#5/4/CROC_1/AE.SQUARROSA (224)/ /OPATA |
| 159 | KULIN | 201 | FGO/USA2111/ /AE.SQUARROSA (658)/3/PRL/SARA/ /TSI/VEE#5/4/ATTILA |
| 160 | CUNNINGHAM | 202 | YAE/AE.SQUAEEOSA (783)/4/GOV/AZ/MUSD/3/SARA/5/MYNA/VUL/JUN |
| 161 | WEAVER | 203 | CROC_1/AE.SQUARROSA (205)/ /KAUZ/3/DHARWAR DRY/4/WBLL1 |
| 162 | MILAN | 204 | AUS 4930.7/2*PASTOR |
| 163 | CHIBIA | 205 | T.TAU.83.2.29/3/PRL/SARA/ /TST/VEE#5/4/CROC_1/AE.SQUARROSA (224)/ /OPATA |
| 164 | PAPAGE M 86 | 206 | CETA/AE.SQUARROSA (327)/ /2*SUNLIN |
| 165 | KAUZ | 207 | CETA/AE.SQUARROSA (327)/ /2*SUNLIN |
| 166 | ANDES39.93 | 208 | T.DICOCCON PI225332/AE.SQUARROS (895/ /WBILL1/3/2*WVLL1 |
| 167 | BAW898 | 209 | T.DICOCCON PI94625/AE.SQUARROSA (372)/ /3*PASTOR |
| 168 | CUMHURIYET 75 | 210 | T.DICOCCON PI225332/AE.SQUARROSA (895)/ /WBLL1/3/2*WBLL1 |
| 169 | GONEN | 211 | T.DICOCCON PI225332/AE.SQUARROSA (895)/ /WBLL1/3/2*WBLL1 |
| 170 | GRANERO INTA | 212 | T.DICOCCON PI94625/SQUARROSA (372)/ /3*PASTOR |
| 171 | INIA F 66 | 213 | CHEN/AE.SQ/ /2*OPATA/3/TRCH |
| 172 | PJ62/GB55 | 214 | CHEN/AE.SQ/ /2*OPATA/3/FINSI |
| 173 | LERMA ROJO 64A | 215 | CHEN/AE.SQ/ /2*OPATA/3/FINSI |
| 174 | MEXIPAK65 | 216 | FRET2/3/CHEN/AE.SQ/ /2*OPATA |
| 175 | MILLALEAU INIA | 217 | CNDO/R143/ /ENTE/MEXI_2/3/AEGILOPS SQUARROSA (TAUS)/4/WEAVER/5/… |
| 176 | OROFEN 60 | 218 | CP18/GEDIZ/3/GOO/ /ALB/CRA/4/AE.SQUARROSA (193)/5/TILHI/6/FRET2 |
| 177 | PAVON F 76 | 219 | ARLIN_1AE.SQUARROSA (1017)/ /ATTILA/3/ATTILA*2/M10 (MUTATED C-306) |
| 178 | PIRSABAK 85 | 220 | ARLIN_1/AE.SQUARROSA (1017)/PARUS/3/TRCH |
| 179 | POTAM S 70 | 221 | 68.111/RGB-U//WARD/3/FGO/4/RABI/5/AE.SQUARROSA(882)/6/ATTILA/10/… |
| 180 | SAFED LERMA | 222 | 68.111/RGB-U//WARD/3/FGO/4/RABI/5/AE.SQUARROSA(882)/6/ATTILA/10/… |
| 181 | SAKHA 69 | 223 | FALCIN/AE.SQUARROSA(312)/3/THB/CEP7780//SHA4/LIRA/4/FRET2 |
| 182 | SALAMANCA 75 | 224 | CROC_1/AE.SQUARROSA(224)/2*OPTATA/3/THB/CEP7780/SHA4/LIRA/4/FRET2 |
|  |  | 225 | ALTAR 84/AE.SQUARROSA (211)/3/CAZO/KAUZ//KAUZ/4/KASO2 |

**Additional file 1; Table S2:** Comparative analysis of environmental factors in two different environments/years using t-test.

| Month | P-value | | | | | | | |
| --- | --- | --- | --- | --- | --- | --- | --- | --- |
|  | Max Temp. (°C) | Min. Temp (°C) | Max RH (%) | Min RH(%) | Monthly R/F (mm) | Seasonal R/F (mm) | Pressure (Hpa) 03 UTC | Pressure (Hpa) 12 UTC |
| Nov. | 0.01 | 0.17 | 0.03 | 0.00 | - | - | 0.25 | 0.19 |
| Dec. | 0.05 | 0.00 | 0.32 | 0.00 | 0.00 | 0.00 | 0.21 | 0.23 |
| Jan | 0.48 | 0.30 | 0.05 | 0.02 | 0.00 | 0.00 | 0.00 | 0.00 |
| Feb | 0.00 | 0.23 | 0.00 | 0.00 | 0.00 | 0.00 | 0.16 | 0.12 |
| March | 0.00 | 0.00 | 0.03 | 0.00 | 0.00 | 0.00 | 0.00 | 0.00 |
| April | 0.01 | 0.23 | 0.15 | 0.37 | 0.00 | 0.00 | 0.37 | 0.16 |
| May | 0.08 | 0.22 | 0.03 | 0.03 | 0.04 | 0.00 | 0.49 | 0.12 |

**Additional File 1: Fig. S1.** The biological meaning of spike layer uniformity (the consistency of the spike distribution in the vertical space) related traits (SLURTs) shown with wheat plants having similar tillers height (a) and different tiller height (b). In (a) only one spike layer can be seen from the vertical perspective (SLN = 1) as spikes per plant have consistent vertical distribution (SLU = 1) and the SLT was identical to one spike length (SL) whereas in (b) two spike layer can be seen from the vertical perspective (SLN = 2) as spikes per plant have inconsistent vertical distribution (SLU = 0.5) and the SLT was identical to two spike length. A detailed description of SLURTs is provided in table 1. Note: In the figure, immature wheat plants are shown just to illustrate the biological meaning of SLURTs. However, data for SLURTs were recorded on mature plants. In the figure scaling is approximate. The figure is based on the publication by Zhao et al. (2019).


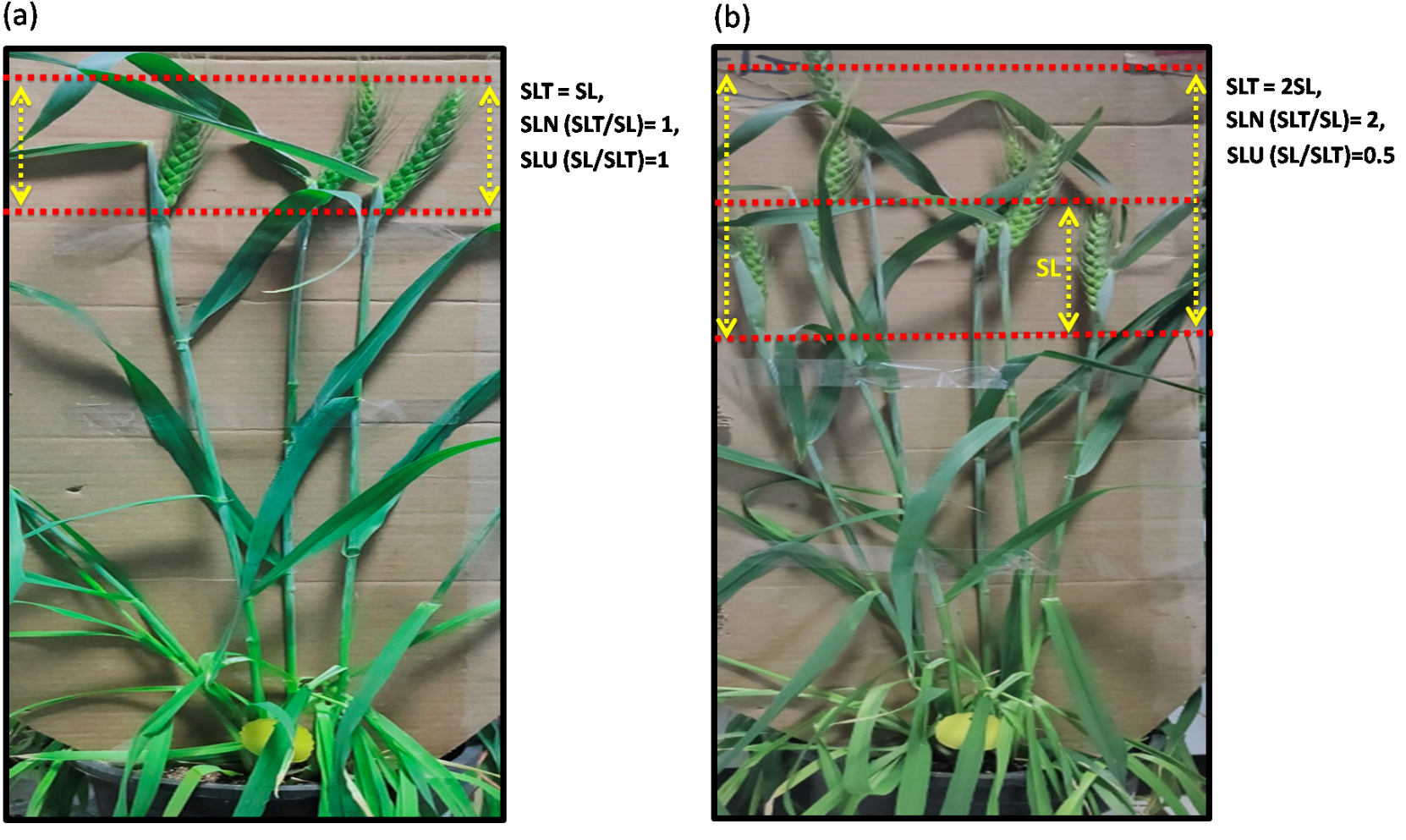


**Additional File 1: Fig. S2.** Pairwise Pearson correlation among the eight SLURTs in E1 and grain yield (GYPP). A single asterisk (*) represents 0.05 level of significance. A double asterisk (**) represents 0.01 level of significance and a triple asterisk (***) represents 0.001 level of significance.


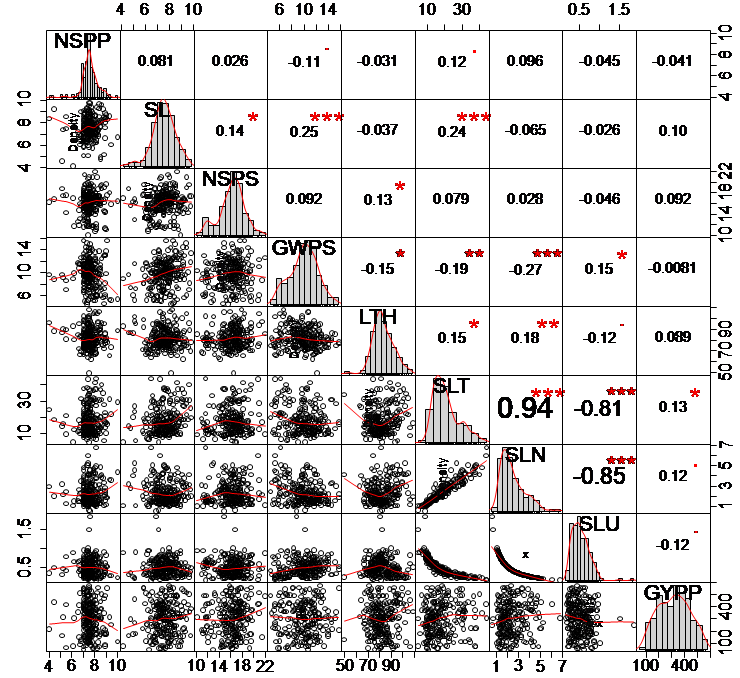


**Additional File 1: Fig. S3.** Pairwise Pearson correlation among the eight SLURTs in E2 and grain yield (GYPP). A single asterisk (*) represents 0.05 level of significance. A double asterisk (**) represents 0.01 level of significance and a triple asterisk (***) represents 0.001 level of significance.


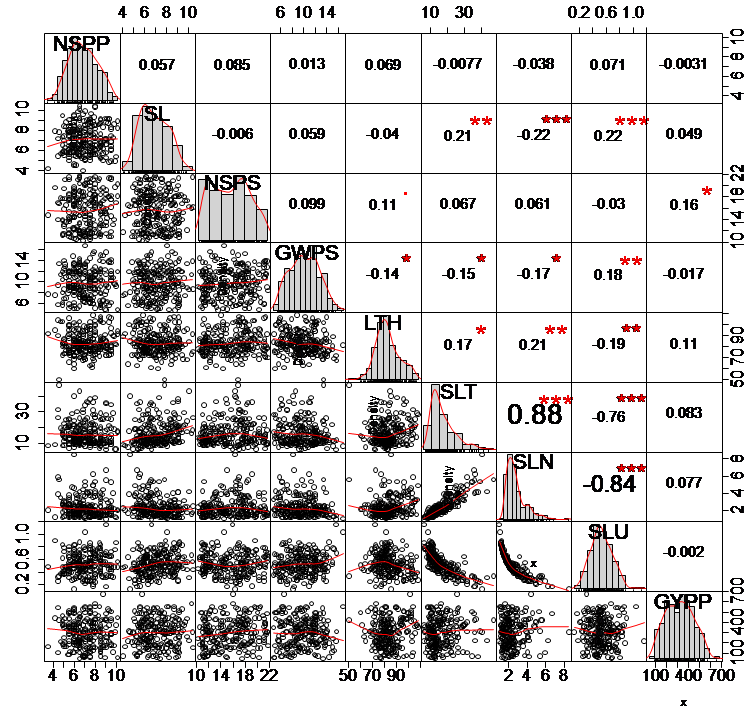


**Additional File 1: Fig. S4.** Pairwise Pearson correlation among the eight SLURTs and grain yield (GYPP) for the combined E1 and E2 environments. A single asterisk (*) represents 0.05 level of significance. A double asterisk (**) represents 0.01 level of significance and a triple asterisk (***) represents 0.001 level of significance.


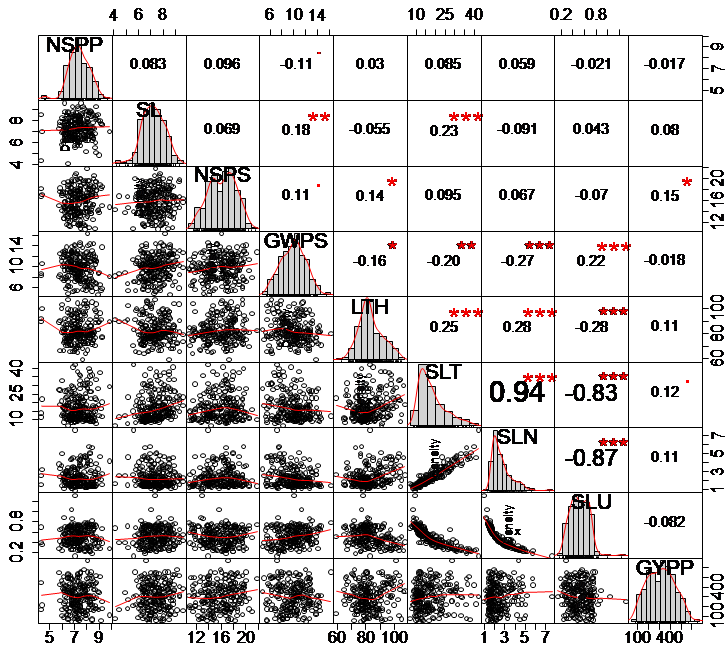


**Additional File 1: Fig. S5.** Manhattan plots obtained after using FarmCPU for eight SLURTs (a) GWPS (b) LTH (c) NSPP (d) NSPS (e) SL (f) SLN (g) SLT (h) SLU under E1 (above numbers) and E2 (below numbers) conditions. Numbers correspond to wheat chromosome.
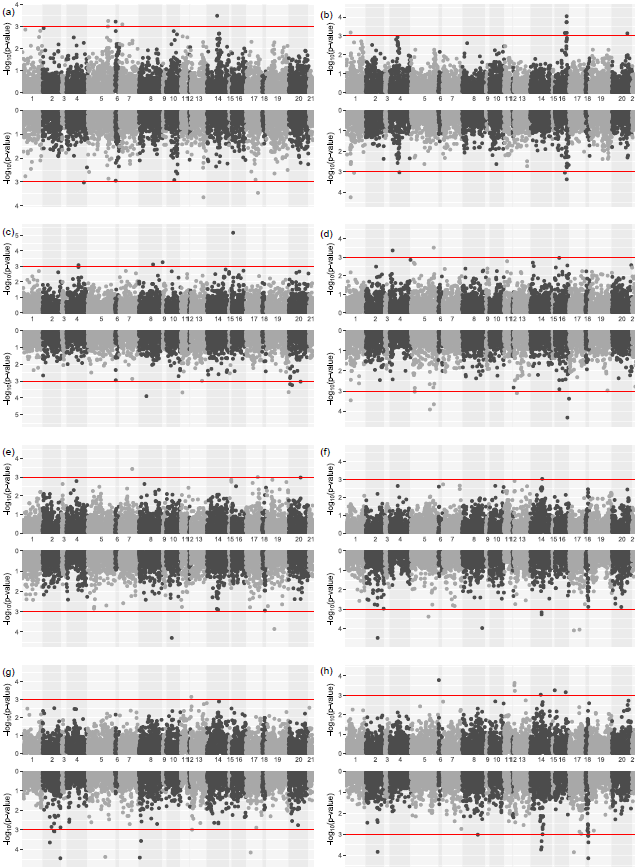


**Additional File 1: Fig. S6.** QQ-plots to visualize the deviation of observed *p* values from expected *p* values (based on null hypothesis) for eight SLURTs (a) GWPS (b) LTH (c) NSPP (d) NSPS (e) SL (f) SLN (g) SLT (h) SLU under E1 and E2 conditions


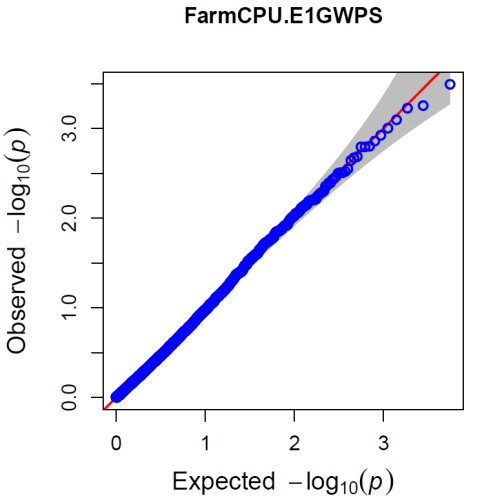

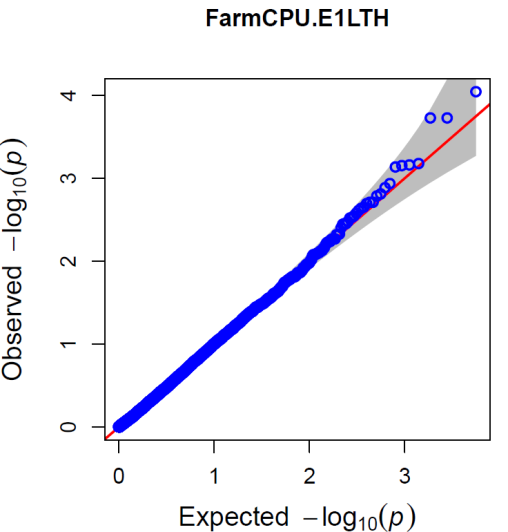

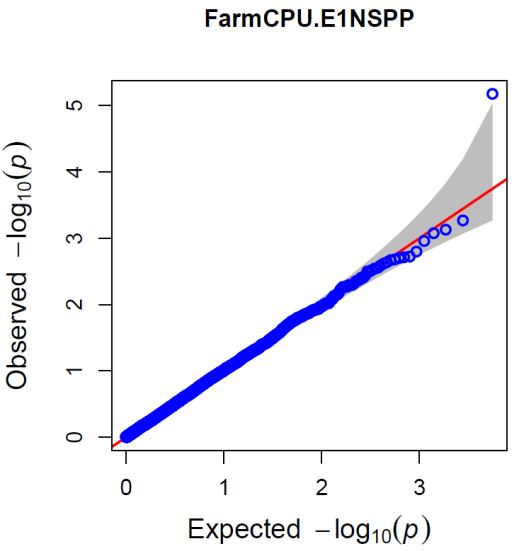

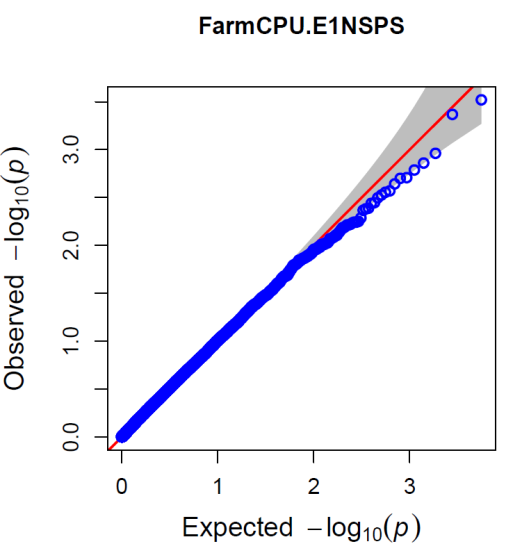

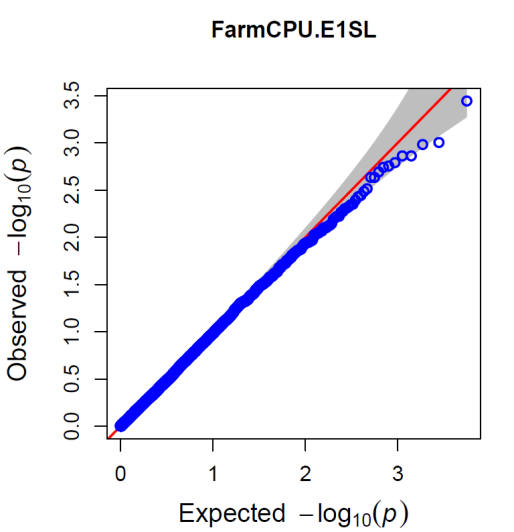

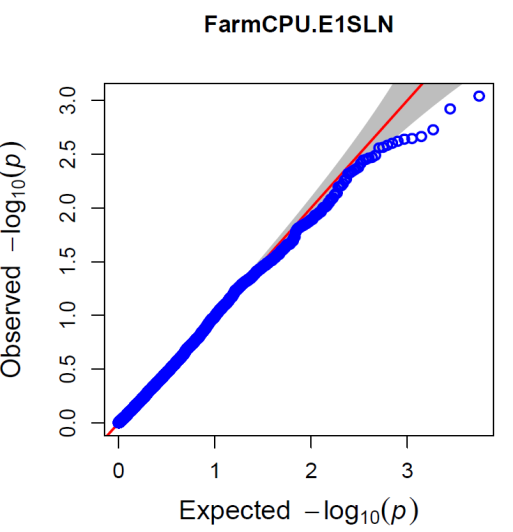

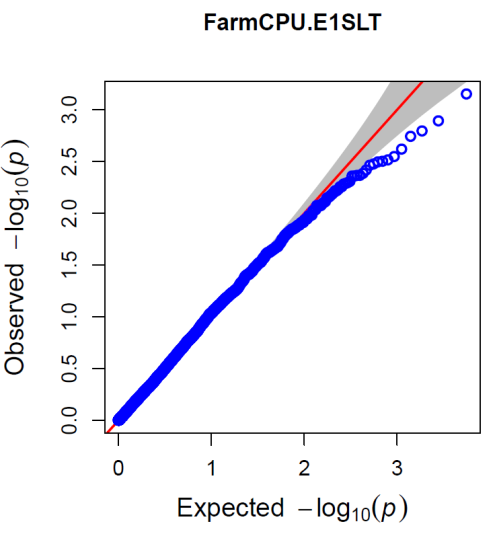

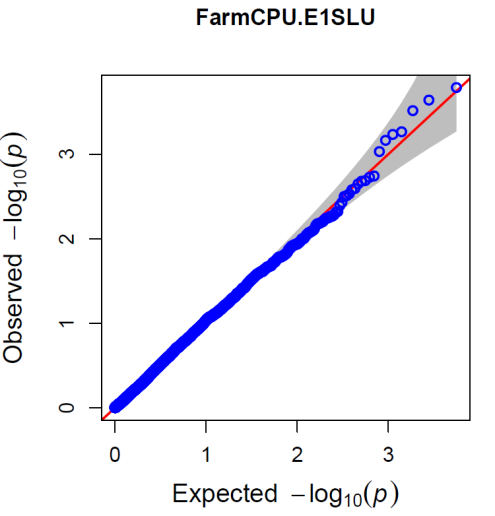


(a)

(b)

(c)

(d)

(e)

(f)

(g)

(h)

**Environment (E1)**


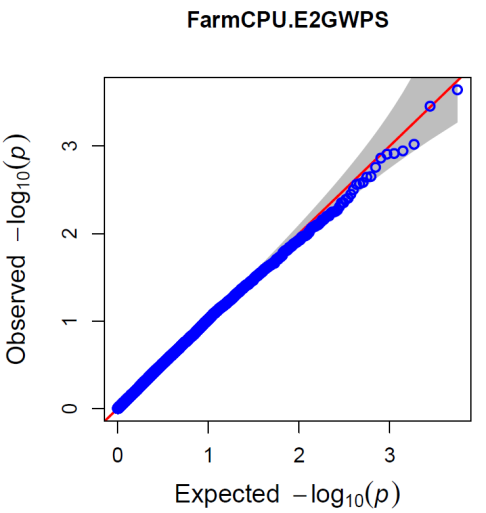

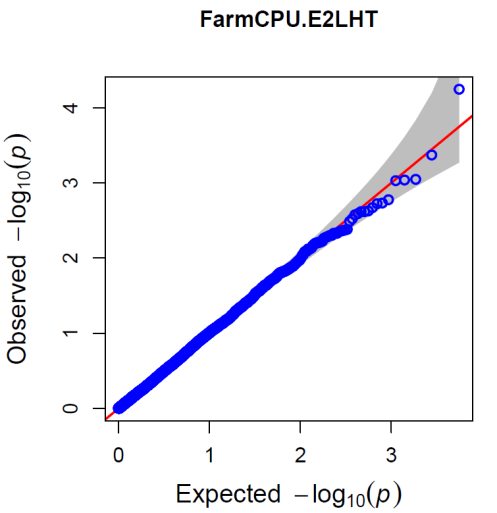

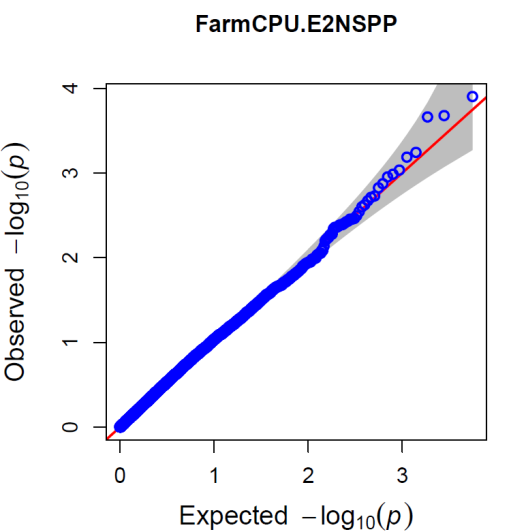

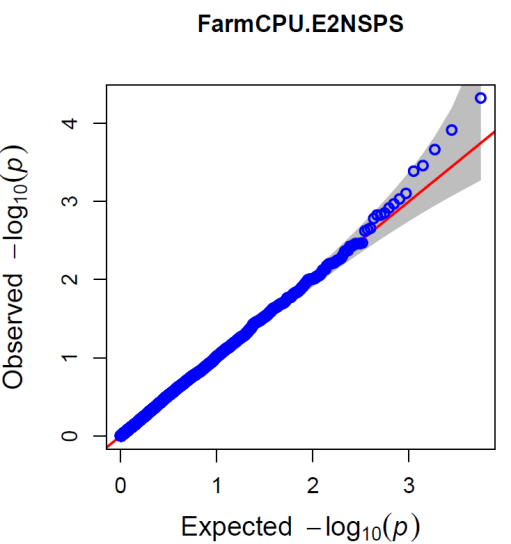

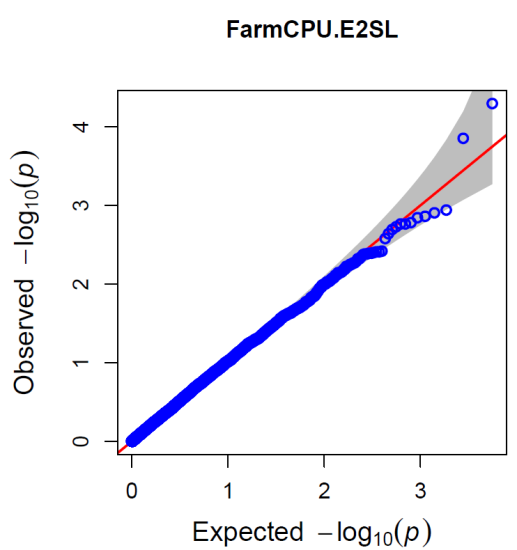

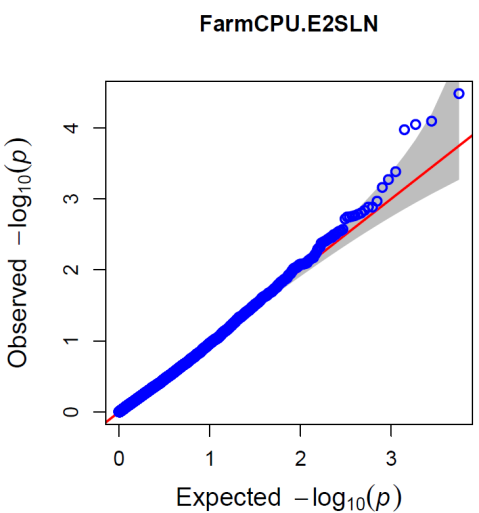

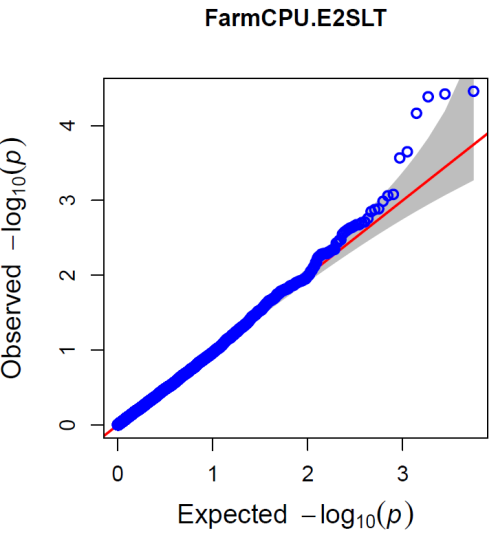

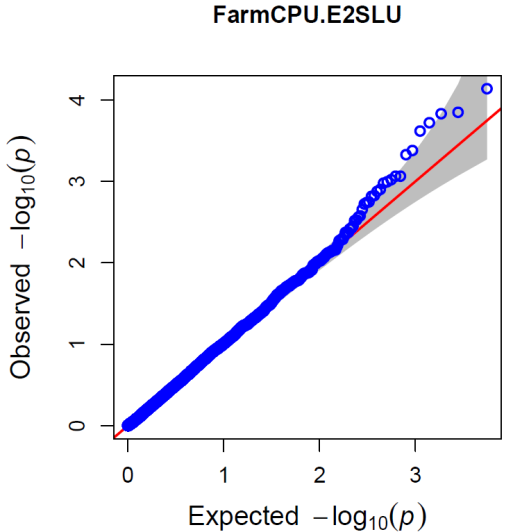


(a)

(b

(c)

(d)

(e)

(f)

(g)

(h)

**Environment (E2)**

**Additional File 1: Fig. S7 (i-v). Comparison of MTAs or MTA groups, detected in the present study, with historical QTLs/MTAs in different chromosomes; MTAs detected in known flanking regions or locations of QTLs/MTAs for same traits or grain yield are depicted in green color and MTAs found near to flanking regions or locations (less than 50 Mb) of QTLs/MTAs depicted in red colour. Flanking markers are shown with the same colour and with the reference details of the previous study. Novel MTAs detected in the present study are presented without any colour. The corresponding physical distances (Mb) of the QTL/MTA regions on each chromosome were obtained by blasting the flanking sequences of markers, depicted on the right side of each figure, to the Chinese Spring RefSeq v1.0. GY; grain yield, TN; tiller number.**


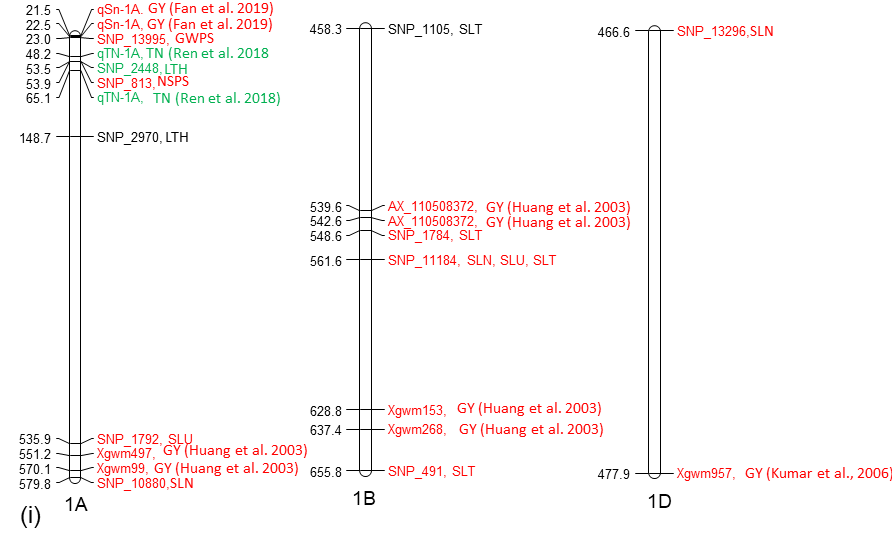


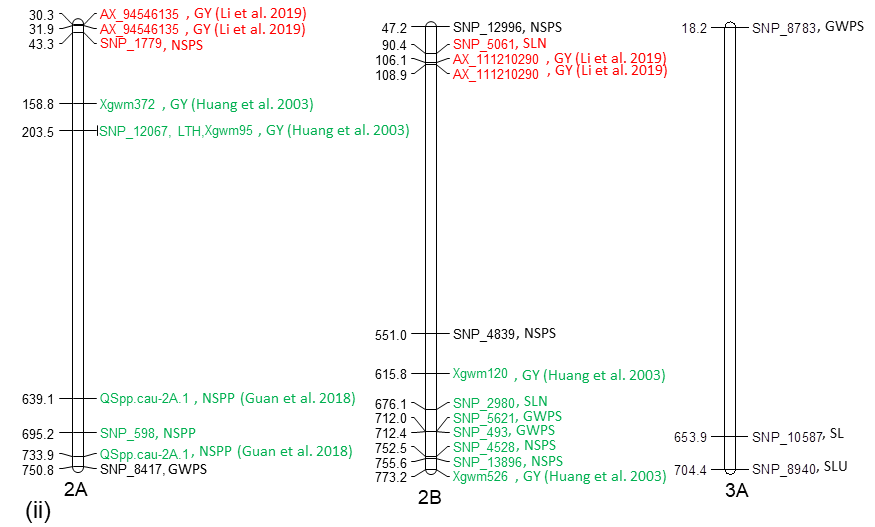


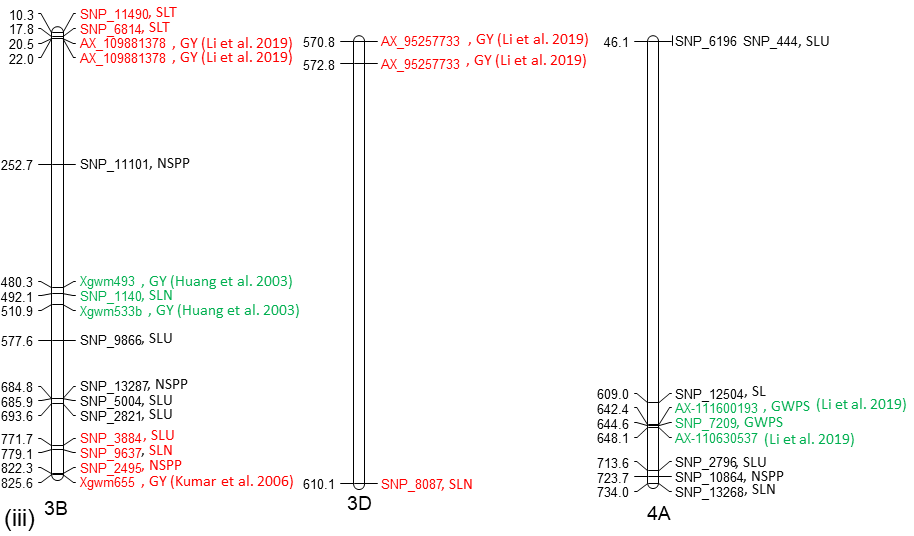


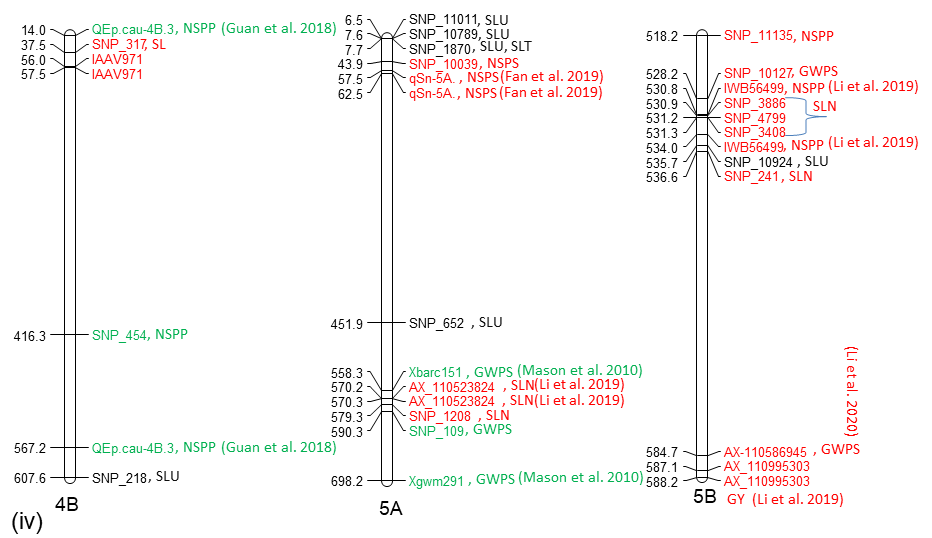


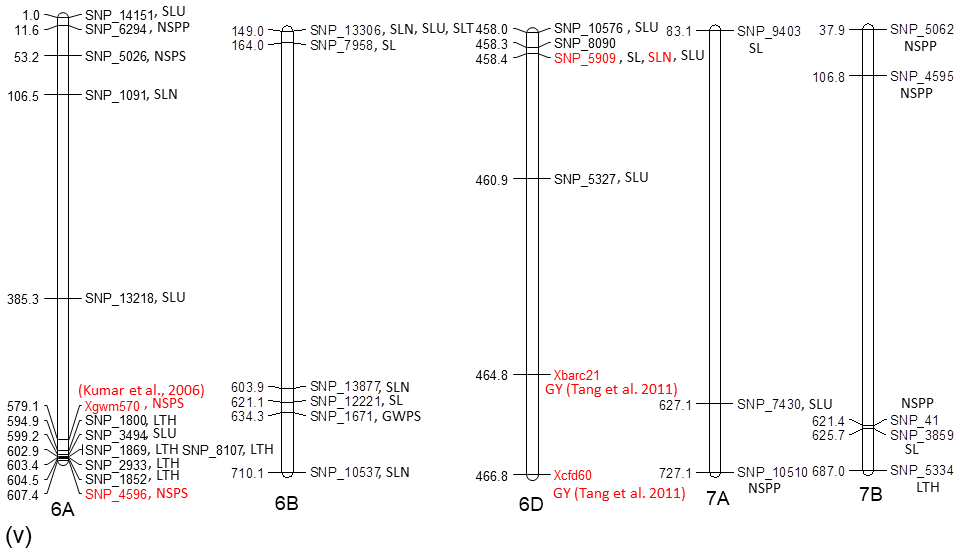

Supplement: Supplementary file 1 — Additional file 1: Table S1: List of 225 spring wheat reference set genotypes. Table S2: Comparative analysis of environmental factors in two different environments/years using t-test. Fig. S1. The biological meaning of spike layer uniformity (the consistency of the spike distribution in the vertical space) related traits (SLURTs) shown with wheat plants having similar tillers height (a) and different tiller height (b). In (a) only one spike layer can be seen from the vertical perspective (SLN = 1) as spikes per plant have consistent vertical distribution (SLU = 1) and the SLT was identical to one spike length (SL) whereas in (b) two spike layer can be seen from the vertical perspective (SLN = 2) as spikes per plant have inconsistent vertical distribution (SLU = 0.5) and the SLT was identical to two spike length. A detailed description of SLURTs is provided in Table 1. Note: In the figure, immature wheat plants are shown just to illustrate the biological meaning of SLURTs. However, data for SLURTs were recorded on mature plants. In the figure scaling is approximate. The figure is based on the publication by Zhao et al. [12]. Fig. S2. Pairwise Pearson correlation among the eight SLURTs in E1 and grain yield (GYPP). A single asterisk (*) represents 0.05 level of significance. A double asterisk (**) represents 0.01 level of significance and a triple asterisk (***) represents 0.001 level of significance. Fig. S3. Pairwise Pearson correlation among the eight SLURTs in E2 and grain yield (GYPP). A single asterisk (*) represents 0.05 level of significance. A double asterisk (**) represents 0.01 level of significance and a triple asterisk (***) represents 0.001 level of significance. Fig. S4. Pairwise Pearson correlation among the eight SLURTs and grain yield (GYPP) for the combined E1 and E2 environments. A single asterisk (*) represents 0.05 level of significance. A double asterisk (**) represents 0.01 level of significance and a triple asterisk (***) represents 0.001 lev [file 12864_2021_7834_MOESM1_ESM.docx]
